# Individual Differences in Frequency and Topography of Slow and Fast Sleep Spindles

**DOI:** 10.3389/fnhum.2017.00433

**Published:** 2017-09-05

**Authors:** Roy Cox, Anna C. Schapiro, Dara S. Manoach, Robert Stickgold

**Affiliations:** ^1^Department of Psychiatry, Beth Israel Deaconess Medical Center Boston, MA, United States; ^2^Department of Psychiatry, Harvard Medical School Boston, MA, United States; ^3^Department of Psychiatry, Massachusetts General Hospital Charlestown, MA, United States; ^4^Athinoula A. Martinos Center for Biomedical Imaging Charlestown, MA, United States

**Keywords:** sleep spindles, individual differences, spatial filter, generalized eigendecomposition, EEG

## Abstract

Sleep spindles are transient oscillatory waveforms that occur during non-rapid eye movement (NREM) sleep across widespread cortical areas. In humans, spindles can be classified as either slow or fast, but large individual differences in spindle frequency as well as methodological difficulties have hindered progress towards understanding their function. Using two nights of high-density electroencephalography recordings from 28 healthy individuals, we first characterize the individual variability of NREM spectra and demonstrate the difficulty of determining subject-specific spindle frequencies. We then introduce a novel spatial filtering approach that can reliably separate subject-specific spindle activity into slow and fast components that are stable across nights and across N2 and N3 sleep. We then proceed to provide detailed analyses of the topographical expression of individualized slow and fast spindle activity. Group-level analyses conform to known spatial properties of spindles, but also uncover novel differences between sleep stages and spindle classes. Moreover, subject-specific examinations reveal that individual topographies show considerable variability that is stable across nights. Finally, we demonstrate that topographical maps depend nontrivially on the spindle metric employed. In sum, our findings indicate that group-level approaches mask substantial individual variability of spindle dynamics, in both the spectral and spatial domains. We suggest that leveraging, rather than ignoring, such differences may prove useful to further our understanding of the physiology and functional role of sleep spindles.

## Introduction

Sleep spindles are prominent rhythmic waveforms expressed by the mammalian brain during non-rapid eye movement (NREM) sleep. In humans, spindles are readily visible in the electroencephalogram (EEG) and are defined as short (~0.5–2 s) bursts of activity in the sigma band (9–16 Hz; but see below). While they are initiated in the thalamus (Steriade et al., 1987), reciprocal interactions between cortex and thalamus shape their duration and amplitude (Contreras et al., 1997; Bonjean et al., 2011). Spindles are a defining feature of light N2 sleep, but also occur during deep N3 sleep where their occurrence is often obscured by large-amplitude ~1 Hz slow oscillations. Moreover, spindles can be present globally or restricted to specific brain regions (Nir et al., 2011; Frauscher et al., 2015; Piantoni et al., 2016), propagate across the cortex (O’Reilly and Nielsen, 2014; Souza et al., 2016), and show complex patterns of interregional synchronization (Cox et al., 2014b; Muller et al., 2016). Functionally, sleep spindles are believed to be involved in processes of plasticity and offline memory consolidation (De Gennaro and Ferrara, 2003; Lüthi, 2013; Rasch and Born, 2013) as evidenced from relations between spindle activity and memory retention (Schabus et al., 2004; Cox et al., 2012; Mednick et al., 2013; Lustenberger et al., 2016).

Although the formal definition treats spindles as an undifferentiated category restricted to the 11–16 Hz range (most commonly 12–14 Hz; Iber et al., 2007), substantial evidence suggests that spindles can be classified as either slow (~10 Hz) or fast (~13 Hz). While it is presently unclear whether slow and fast spindles serve distinct functional roles (Tamaki et al., 2008, 2009; Barakat et al., 2011; Cox et al., 2014a; Hoedlmoser et al., 2014; Rihm et al., 2014; Fang et al., 2017), they are associated with different hemodynamic sources (Schabus et al., 2007), respond differently to pharmacological interventions (Ayoub et al., 2013), preferentially occur in distinct phases of the slow oscillation (Mölle et al., 2011; Cox et al., 2014b; Staresina et al., 2015; Klinzing et al., 2016), and are dissociated in terms of heritability (Purcell et al., 2017). Slow and fast spindles also have distinct EEG topographical distributions, with slow spindles having a more frontal expression and fast spindles occurring mostly centrally and parietally (Werth et al., 1997; Zeitlhofer et al., 1997). Importantly, while the slow spindle band roughly corresponds to the waking alpha range (8–12 Hz), individuals’ alpha activity is typically slower (Kokkinos and Kostopoulos, 2011) and is defined by a distinct posterior topographical distribution, suggesting these rhythms reflect distinct phenomena. Although differential slow/fast spindle topographies are well known at the group-level, little is known about the consistency of these topographical patterns across individuals. However, assessing this variability is an important step towards a full understanding of the dynamics of slow and fast sleep spindle activity within and across individuals.

Separating slow and fast spindles is nontrivial, in part because of the diversity of spectral definitions used by different groups for spindle detection. A non-exhaustive search of the literature reveals demarcation frequencies between slow and fast spindles placed at 12, 13, 13.5 and 14 Hz (Schabus et al., 2007; Barakat et al., 2011; Mölle et al., 2011; Holz et al., 2012; Ayoub et al., 2013; Wamsley et al., 2013). Similarly, the lower boundary of slow spindles has been set anywhere from 8 Hz to 12 Hz (Barakat et al., 2011; Mölle et al., 2011; Holz et al., 2012; Ayoub et al., 2013; Wamsley et al., 2013), and the higher boundary of fast spindles at 15 or 16 Hz (Tamaki et al., 2008; Ayoub et al., 2013). Similar variability exists for studies not further differentiating between slow and fast spindles. Clearly, this situation hinders comparison across studies.

The issue is exacerbated further by considerable variability in spindle frequency across individuals (De Gennaro et al., 2005; Ujma et al., 2015). Subject-specific spindle frequencies are typically determined from peaks in the power spectrum. However, slow and fast sigma peak frequencies are not confined to well-separated frequency ranges but form overlapping distributions at the group level (Ujma et al., 2015). Thus, even the demarcation line best separating slow and fast sigma peaks at the group-level likely results in incorrect classification of spindle activity for some subjects and suboptimal separation for many others.

Together, individual differences in peak sigma frequencies and variable spectral spindle criteria distort the correspondence between the oscillatory phenomena of interest and the measured activity used for subsequent analysis. This problem affects approaches investigating sigma power as a proxy for spindle activity (Achermann and Borbély, 1998), as well as spindle detection algorithms based on band-pass filters and amplitude thresholds (e.g., Ferrarelli et al., 2007; Cox et al., 2014a).

To avoid these issues, approaches targeting subject-specific spindle frequencies have been developed (Gottselig et al., 2002; Bódizs et al., 2009; Mölle et al., 2011; Adamczyk et al., 2015; O’Reilly et al., 2015; Ujma et al., 2015). However, while fast sigma peaks are typically prominent, slow sigma peaks are not always discernible, even at frontal channels where slow spindle activity is generally most pronounced (Mölle et al., 2011). This may reflect that slow and fast spindle topographies, while distinct, still show considerable overlap at both the level of sensors and underlying generators (Klinzing et al., 2016). Moreover, as we will demonstrate, individual differences in spindle topography limit the effectiveness of selecting a single channel for slow sigma peak detection. Finally, slow spindles have been observed to express marked shifts of ~1 Hz between sleep stages, from faster in N2 to slower in N3 (Mölle et al., 2011). In sum, available methods to separate spindle classes do not always succeed and can lead to ambiguous results depending on what sleep stage is examined.

To overcome these difficulties, we introduce a novel approach to determine individualized slow and fast sigma frequencies in a data-driven fashion. The current report is organized into three main parts. In part one (Channel-Based Analyses), we describe individual differences in the NREM power spectrum, focusing on the difficulties of detecting subject-specific sigma peaks from both channel-averaged and single-channel spectra. In part two (Component-Based Analyses), we detail a spatial filtering approach to facilitate isolation of subject-specific slow and fast sigma frequencies. We show that spectra derived from spatially filtered data allow for slow peak detection in more individuals and with less ambiguity than channel-based spectra. In part three (Topographical Analyses), we characterize topographical aspects of slow and fast sleep spindle expression in N2 and N3, both to validate our sigma peak separation method, and to examine spatial aspects of spindles not yet described. In particular, we investigate the commonalities, individual differences, and cross-night stability of spatially organized spindle activity, as well as topographical differences between several often-used metrics of spindle activity. Part three will be particularly relevant to those interested in the implications of these methods for topographical spindle dynamics, and can be read separately from the first two parts. Combined, our results demonstrate the utility of our spindle separation approach and yield important new insights regarding the nature and variability of topographical sleep spindle dynamics.

## Materials and Methods

### Protocol and Participants

The current study utilizes two consecutive nights of full-night EEG data from 28 healthy individuals (age: 29.7 ± 6.0; 21 males, 7 females). These data were acquired as part of a double-blind, placebo-controlled, cross-over study of eszopiclone in schizophrenia patients. Only the placebo nights of the control group are considered in the present report.

The study protocol was approved by the Partners Human Research Committee. All subjects gave written informed consent in accordance with the Declaration of Helsinki and were compensated monetarily for their participation. Participants had no personal history of mental illness as confirmed by screening with the SCID-Non-Patient Edition (First et al., 2002). Furthermore, they reported no diagnosed sleep disorders, treatment with sleep medications, history of significant head injury or neurological illness, or history of substance abuse or dependence within the past 6 months. Upon completion of a pre-treatment visit to complete informed consent and undergo clinical and cognitive assessments, subjects received an actiwatch to wear from study enrollment to completion.

Subjects were randomly assigned to one of two treatment orders, placebo first or eszopiclone first, with a week in between the two treatment visits. Each of the two treatment visits consisted of two consecutive nights of polysomnography (PSG) monitoring at the Clinical Research Center of Massachusetts General Hospital. The first night of each visit served as a baseline night, while on the second night participants were trained for 12 min on a finger tapping Motor Sequence Task (Walker et al., 2002) 1 h prior to their usual bedtime. On both nights of the placebo visit, participants received placebo at 10 PM. Lights were turned off at 10.30 PM and participants were allowed to sleep for up to 9.5 h until they were woken up at 8 AM. As no statistically significant differences in sleep architecture or spindle parameters were found between the baseline and learning nights, we focused our analyses on the first night and used the second night for validation and replication.

### Data Acquisition and Preprocessing

PSG was collected using 62-channel EEG caps (Easycap GmbH, Herrsching, Germany) with channel positions in accordance with the 10-20 system. Additionally, two single cup electrodes were placed on the mastoid processes, two around the eyes for electrooculography, two on the chin for electromyography, and a reference electrode was placed on the forehead. An AURA-LTM64 amplifier and TWin software were used for data acquisition (Grass Technologies, Warwick, RI, USA). Impedances were kept below 25 kΩ and data were sampled at 400 Hz with hardware high-pass and low-pass filters at 0.1 Hz and 133 Hz, respectively.

Sleep staging was performed in TWin using a limited number of channels with a contralateral mastoid reference on 30 s epochs according to standard criteria (Iber et al., 2007). Initial processing of multi-channel EEG data was performed in BrainVision Analyzer 2.0 (BrainProducts, Germany). All EEG channels were band-pass filtered between 0.3 Hz and 35 Hz and notch filtered at 60 Hz. Channels displaying significant artifacts for more than 30 min of the recording were interpolated with spherical splines. EEG data were then re-referenced to the average of all EEG channels. Upon visual inspection, epochs containing artifacts were removed. To remove artifacts we used independent component analysis (ICA) with the Infomax algorithm (Makeig et al., 1997). For each night and individual, remaining epochs were concatenated separately for the two sleep stages, resulting in 176 ± 61 (mean ± SD) and 211 ± 49 min of available N2 for the two nights, and 82 ± 44 and 85 ± 30 min of N3.

All subsequent processing steps were performed in Matlab (The Mathworks, Natick, MA, USA), using custom routines and several freely available toolboxes including EEGlab (Delorme and Makeig, 2004) and Fieldtrip (Oostenveld et al., 2011). After removal of non-EEG channels and the mastoids, leaving 58 channels for analysis, we applied a surface Laplacian filter to each record (Perrin et al., 1989), as implemented in the CSD toolbox (Kayser and Tenke, 2006). This approach served two purposes. First, the Laplacian renders data reference-free, thereby avoiding common interpretational issues related to the choice of reference. Second, this approach decreases the effects of volume conduction and accentuates local aspects of neural processing, thereby providing enhanced spatial precision for topographical analyses (Cohen, 2014; Tenke and Kayser, 2015). While the Laplacian is a spatial filter, we emphasize it is not the spatial filtering approach that we employ to separate slow and fast sigma peaks.

### Power Spectra and Peak Detection

After the Laplacian transformation, we determined the power spectrum for every epoch on every channel. In order to minimize the typical 1/f scaling of the spectrum, we obtained power estimates not from the Laplacian-transformed time series, but from its temporal derivative. This approach essentially multiplies power at every frequency bin by its frequency, thus counteracting the 1/f trend and allowing for easier detection of spectral peaks relative to surrounding frequencies (Sleigh et al., 2001). Using the Laplacian-derivatives, we estimated power spectral density for each epoch using Welch’s method with 5 s windows and 50% overlap. We then normalized every electrode’s power spectrum during both N2 and N3 by dividing the spectrum by that electrode’s average power in the 0–4 Hz band across all N2 epochs. This normalization step, based on a common baseline for N2 and N3, enables direct comparisons between sleep stages.

To determine global spectra, single-epoch spectra were averaged across all channels, before averaging across epochs, separately for N2 and N3. For visualization and peak detection, each individual’s 0–20 Hz spectra were rescaled between the minimum and maximum values in that range. Spectral peaks were detected using the Matlab *findpeaks* function with a minimum prominence setting of 0.01, where the prominence of a peak indicates how much a peak stands out as a function of both its intrinsic amplitude and its location relative to other peaks. For our data, this setting corresponded to a very liberal detection threshold.

In addition to channel-averaged spectra, we determined spectra for two selected channels (frontal: Fz; parietal: Pz). We selected these channels based on evidence of the relative predominance of slow and fast spindles (Tamaki et al., 2008; Ayoub et al., 2013). Single-channel spectra were averaged across epochs, again separately for N2 and N3 and both nights. Automated spectral peak detection was performed as before.

### Slow and Fast Sigma Peak Separation via Spatial Filters

In order to isolate each individual’s slow and fast sigma activity in data-driven fashion, we created linear spatial filters maximally enhancing slow vs. fast sigma activity and vice versa. We then applied these filters to the multi-channel EEG time series to obtain a set of component time series that we analyzed in the frequency domain. The spatial filters were defined by eigenvectors extracted from covariance matrices, similar to principal component analysis (PCA). In a spatial filtering context, the PCA procedure operates on a single channel-by-channel covariance matrix and produces eigenvectors pointing in orthogonal directions that explain decreasing amounts of variance. This approach, together with conceptually related ICA techniques, can be conceptualized as a “blind” source separation procedure, in that resulting components are not necessarily physiologically meaningful. In contrast, generalized eigendecomposition (GED) operates on two separate covariance matrices to find eigenvectors maximally differentiating the two. This may be viewed as a “guided” source separation procedure that spatially separates signal elements according to user-defined criteria. In our case, we constructed one covariance matrix from slow sigma-filtered data and one from fast sigma-filtered data. The GED approach has been used in various electrophysiological contexts, typically to maximize spectral power in one frequency band relative to broadband activity (Nikulin et al., 2011; de Cheveigné and Arzounian, 2015; Cohen and Gulbinaite, 2017; Cohen, 2017). We here extend this notion by directly contrasting activity in two adjacent narrow-band ranges.

In detail, we first band-pass filtered the Laplacian-transformed EEG separately in the slow (9–12 Hz) and fast (12–16 Hz) sigma ranges. We used Hamming-windowed finite impulse response filters (EEGlab: *pop_firrws*) with a high filter order (13,200) to create steep, narrow filters that have minimal overlap between the two passband ranges. After subtracting each filtered channel’s mean amplitude, we determined the “slow” covariance matrix **S** and the “fast” covariance matrix **F**, both of size 58 × 58 electrodes. If we designate **S** as the matrix whose signal we wish to accentuate and **F** as the matrix with the “noise” we wish to suppress, the eigendecomposition problem can be written as **SW** = **WFΛ**, where **W** is a matrix of eigenvectors and **Λ** is a diagonal matrix of eigenvalues. In Matlab, **W** and **Λ** can be found via *[W,L] = eig(S,F)*. The column in **W** with the highest corresponding eigenvalue then corresponds to the eigenvector that maximally enhances slow relative to fast sigma activity. Conversely, the eigenvector with the lowest eigenvalue has the opposite effect, maximizing fast relative to slow sigma power. Detailed treatments of the derivation of these equations can be found elsewhere (Nikulin et al., 2011; de Cheveigné and Arzounian, 2015; Cohen, 2017; Cohen and Gulbinaite, 2017).

Although the most useful eigenvectors generally have relatively high and low eigenvalues, it is not known *a priori* which eigenvectors will yield the best results. We therefore multiplied the multi-channel, raw, broadband, Laplacian-transformed, EEG with the full matrix **W**, resulting in a time series of 58 components (where each component reflects a unique spatial weighting across all channels). These component time series, in turn, were transformed to the frequency domain. Similar to channel-based power spectra, we first took the temporal derivative of the multi-component time series, and then estimated power spectral density using Welch’s method with 5 s windows and 50% overlap. We again took the temporal derivative approach to reduce 1/f noise, and make channel- and component-based peak detection as similar as possible. However, we note that component peak location was not noticeably influenced by the temporal derivative approach. Resulting component spectra were visualized and the first slow and the first fast component with peaks of sufficient quality were selected based on visual inspection. Typically, clear spectral sigma peaks were visible within the first 10 (for slow sigma) or last 10 (for fast sigma) components, with several components peaking at exactly the same frequency. Although manual component selection is time-consuming and ultimately subjective, we note this process is akin to routine manual selection of ICA components for removal or analysis. Importantly, an individual’s components were selected without cross-referencing them against that individual’s channel-based spectra, guarding against experimenter bias. Moreover, as we will demonstrate, components independently selected for N2 and N3 and for the two nights showed a close correspondence in peak frequency for each subject; frequencies of these manually selected components were determined with an automated peak detection algorithm as before. Supplementary Matlab code demonstrates how to implement the GED analysis for several example sleep recordings (see “Data and Software Sharing” Section).

We note that the spectral bands we used here for temporal filtering (9–12 and 12–16 Hz) are slightly different from the ones we later adopt as approximate slow (9–12.5 Hz) and fast (12.5–16 Hz) spindle ranges based on the distribution of sigma peak locations across individuals. However, we determined in several subjects that shifting initial filter ranges does not affect frequencies of subsequently identified component peaks by more than 0.2 Hz (Supplementary Discussion).

We defined an individual’s slow or fast sigma range as a 1.3 Hz window centered on his or her average sigma peak frequency across nights and sleep stages. This width was a compromise between a sufficiently broad range to capture small within-subject differences in peak frequency across sleep stages and nights, and sufficiently narrow to have non-overlapping slow and fast windows for as many individuals as possible. An additional consideration here was that for subsequent spindle detection (see “Spindle Detection” Section), data needs to be filtered in each individual’s slow or fast sigma range. Thus, we also inspected each individual’s slow and fast sigma filters’ frequency response used for spindle detection. These inspections assisted both in arriving at the 1.3 Hz sigma ranges and in deciding which subjects to remove due to insufficient separation of the slow and fast ranges. Based on these considerations, we opted to exclude three subjects from topographical analyses involving slow spindles, because their N2 and N3 slow sigma peaks differed by more than 0.7 Hz. Additionally, we excluded two subjects whose slow and fast sigma filters still overlapped from all topographical analyses (including one who had already been excluded for slow spindle analysis). Of note, channel and component spectra for one of these to-be-removed subjects (S4) are shown in Figures 2C,G. Thus, these additional exclusions were not due to an inability to resolve closely spaced peaks with the GED component approach, but stemmed from an inability to adequately separate them with filter widths of 1.3 Hz.

**Figure 1:**
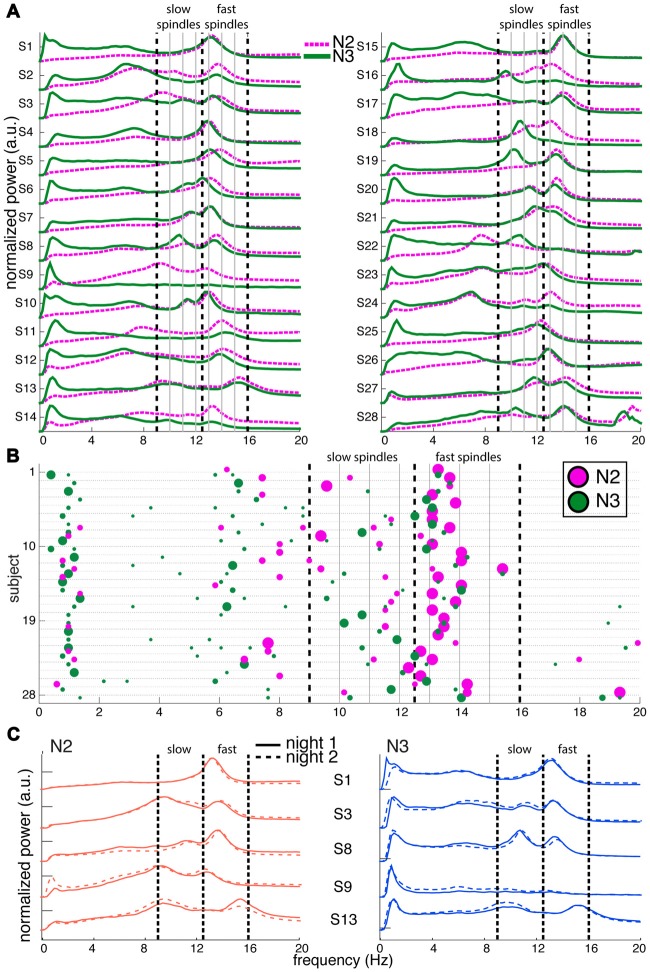
Variability of channel-averaged spectra and spectral peak locations in N2 and N3. **(A)** Individual subjects’ normalized spectra averaged across all channels are shown for N2 (magenta) and N3 (green) from night 1. For visualization, spectra have been rescaled to have the same amplitude range for every subject. Thick vertical dashed lines indicate slow and fast sigma boundaries at 9, 12.5 and 16 Hz. Thin solid vertical lines spaced at 1 Hz intervals assist in evaluating how alternative spectral definitions would partition sigma activity into slow and fast categories. **(B)** Spectral peak frequencies in N2 and N3 from night 1, corresponding to panel **(A)**. Each row represents an individual and colored dots indicate the location of spectral peaks for N2 (magenta, above line) and N3 (green, below line). Size of dots is proportional to peak prominence. Note the large variability in peak frequencies in the sigma range and the absence of clear slow sigma peaks for many individuals. In contrast, peaks in the 0.5–2 Hz range are highly consistent across individuals during N3. Vertical lines as in **(A)**. **(C)** Normalized and rescaled channel-averaged N2 (left, orange) and N3 (right, blue) spectra of five example subjects from night 1 (solid) and night 2 (dashed). Individual differences in spectral shape are highly stable across nights. Vertical lines as in **(A)**.

**Figure 2:**
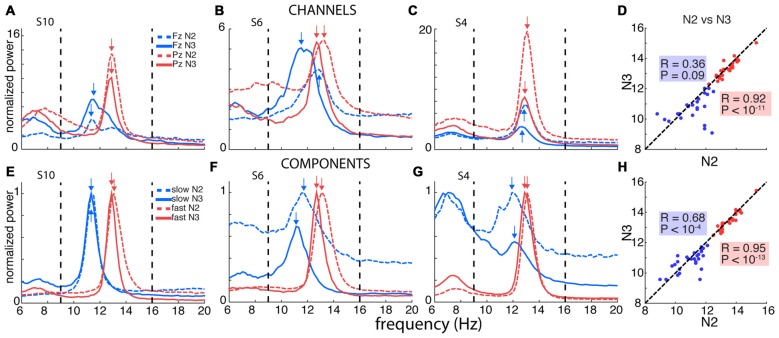
Channel- and component-based spectral peaks. **(A–C)** Single-channel spectra for three selected subjects for frontal channel Fz (blue) and posterior channel Pz (red), for N2 (dashed) and N3 (solid). Power obtained using derivative of Laplacian-transformed time series, and normalized to average 0–4 Hz power during N2. **(D)** Correspondence between channel-based peaks identified during N2 and N3, calculated separately for Fz and Pz. **(E–G)** Component-based spectra for same individuals as in panels **(A–C)**. Spectra scaled between 0 and 1 to account for varying amplitudes of different components. **(H)** Correspondence between component-based peaks identified during N2 and N3, calculated separately for slow and fast components. Correlation for slow sigma peaks is much greater compared to channel-based peak selection in **(D)**, indicating more stable estimates of underlying oscillatory frequency.

### Topographical Cluster Analyses

For topographical analyses of sigma power, we averaged, per epoch and electrode, power across the 1.3 Hz frequency range corresponding to each subject’s individualized sigma peaks, before averaging across epochs. This was done separately for N2 and N3, and for each night. Permutation-based statistical analyses on topographical data (sigma power, as well as spindle density and spindle amplitude derived from automated spindle detection) were performed with Fieldtrip using cluster correction (Maris and Oostenveld, 2007). In several sets of analyses, we compared N2 and N3, and slow and fast spindle topographies for different spindle metrics. Using 1000 iterations, the paired samples *t* statistic, a *clusteralpha* value of 0.1, and a significance threshold of 0.05, clusters were deemed significant at *P* < 0.025 for two-sided testing.

### Spectral and Topographical Similarity

We determined the within-subject similarity of each individual’s power spectra (e.g., between N2 and N3, between nights), and the topographical correspondence of each individual’s spindle expression (e.g., between slow and fast spindles, between N2 and N3) in conceptually and analytically similar ways. First, we calculated the Pearson correlation coefficient between a subject’s two relevant spectra, or two relevant topographies. For spectra, values were normalized power estimates at every frequency bin from 0 Hz to 20 Hz (103 bins). For topographies, values were spindle activity estimates (e.g., sigma power) at each of the 58 electrodes. This yielded, across subjects and for each comparison, a set of *N* correlation coefficients and associated *P* values (where *N* is the number of individuals included in the particular analysis). To assess these results at the group-level, we performed a one-sample *t* test comparing the set of *N* correlation coefficients to zero. In addition, we adjusted the set of *N P* values for multiple comparisons using the False Discovery Rate (Benjamini and Hochberg, 1995) and report the percentage of subjects showing above-chance similarity of spectral or spatial profiles.

Importantly, while within-subject correlation coefficients provide a useful index of “absolute” spectral or topographical similarity, they ignore how similar spectral or topographical profiles are across individuals. For example, average within-subject, cross-night correlation values of 0.8 do not indicate meaningful individual stability if average between-subject correlations are also 0.8. Conversely, within-subject correlations of only 0.4 signal substantial individual stability of spectra or topographies if between-subject correlations are only 0.2.

Second, therefore, we asked how well an individual’s spectral or topographical pattern would allow us to differentiate that subject from other subjects. To this end, we trained, for each comparison of interest, a *k*-nearest neighbor classifier (Cover and Hart, 1967) on all subjects’ spectral/topographical patterns in one condition (e.g., night 1), and tested it on unseen data from the other condition (e.g., night 2). This is implemented in Matlab as *fitcknn*. We set *k* = 1, and used the correlation distance (1 − Pearson correlation) as the distance metric. In essence, this means each unseen test record is labeled as the one it is most highly correlated with from the training set. Classifier performance was calculated as the proportion of test cases that were assigned the correct label, and classifier significance was assessed using binomial tests. Thus, classifier performance provides an index of whether—and how much—spectral or topographical patterns are more stable within than across subjects, thereby complementing the within-subject similarity estimates afforded by Pearson correlation.

### Spindle Detection

Individual sleep spindles were detected with an automated algorithm adapted from one we (RC) employed earlier (Cox et al., 2012, 2014a). For each subject and each channel, and separately for the slow and fast sigma ranges, the Laplacian-transformed signal was zero-phase band-pass filtered in that subject’s frequency range of interest. Specifically, we used Matlab’s *firls* function to design steep filters of order 3000 with a 1.3 Hz passband and 0.5 Hz transition zones around the individualized center frequency. The sigma envelope was calculated as the magnitude of the Hilbert-transformed filtered signal, and was smoothed with a 200 ms moving average window. Whenever the envelope exceeded an upper threshold a potential spindle was detected. Crossings of a lower threshold before and after this point marked the beginning and end, respectively, of the spindle. Start and end points were required to be at least 400 ms and no more than 3000 ms apart, similar to other automated detectors (Ramanathan et al., 2015). Per channel, thresholds were set at the average N2 smoothed sigma amplitude envelope + 3 SDs (upper), and + 1 SD (lower). Spindle events were discarded whenever power in any 20–80 Hz frequency bin exceeded that of any frequency bin in that individual’s sigma range (suggesting a broadband power increase rather than band-specific spindle), or when the spindle’s average amplitude envelope was >4 SD above the mean (indicative of an outlier). We calculated separate threshold settings for slow and fast spindles to optimally adapt to differences in slow and fast sigma amplitude. For both slow and fast spindles, the same N2-based thresholds were used across N2 and N3 to prevent confounding by different levels of sigma power across these sleep stages. We then determined spindle density (spindles per minute) and the mean peak amplitude of spindle events for each combination of channel, sleep stage and spindle class.

### Data and Software Sharing

Open source Matlab code (available at https://doi.org/10.6084/m9.figshare.4905677) demonstrates how to implement the GED analysis for several example sleep recordings. Sharing of de-identified data was exempted from ethical approval and was covered by the informed consent procedure.

## Results

### Channel-Based Analyses

We first describe in some detail individual differences of NREM spectra in general, and of sigma activity in particular. While previous reports have already described several aspects of this variability (De Gennaro et al., 2005, 2008; Lewandowski et al., 2013; Ujma et al., 2015), we here revisit this issue qualitatively and quantitatively in order to make the motivation for a novel slow/fast spindle separation approach explicit. We examine spectral properties from both channel-averaged and single-channel (frontal and parietal) perspectives and demonstrate the difficulties of both employing fixed slow/fast spindle ranges and of utilizing channel-based data to define subject-specific frequency ranges. Readers already familiar with these issues may skip ahead to *Component-Based Analyses* where we introduce an improved spindle separation approach based on spatial filters.

#### Channel-Averaged Spectra

To depict oscillatory activity across the cortex, we averaged 58-channel EEG spectra across all channels for every 30 s epoch, and then across epochs. Figure 1A shows these channel-averaged spectra from night 1 for all 28 subjects during both N2 (magenta) and N3 (green) in the 0–20 Hz range. Figure 1B shows each individual’s detected N2 and N3 peak locations as correspondingly colored dots with surface areas proportional to each peak’s prominence. In both panels **(A,B)**, thick dashed vertical lines placed at 9, 12.5 and 16 Hz indicate boundaries that best separate putative slow and fast sigma peaks across subjects. These boundaries are based on careful inspection of peak frequency distributions in the channel averaged-spectra, as well as the component-based spectra we present later, and agree well with those used by some other groups (Mölle et al., 2011; Ujma et al., 2015). Nonetheless, the overlap between the chosen slow sigma range and classical alpha band activity is potentially problematic. We justify interpreting dynamics in this band as spindle activity in the “Discussion” Section.

There was high variability in spectral profiles between subjects, especially in the prominence and number of visible peaks in the sigma range. Using a peak detection method with liberal sensitivity, we could identify one or more peaks in the broader 9–16 Hz range for all 28 subjects in both N2 and N3. In N2, we observed 13 individuals with just one peak, 14 with two, and one subject with three (S13), although there was typically one peak that was much more pronounced than the others. In N3, 12 individuals had just one identifiable peak, and 16 had two. Peaks in the fast sigma range (12.5–16 Hz) were relatively pronounced during both N2 (*n* = 27) and N3 (*n* = 24; including two subjects who had N3 peaks exactly at 12.5 Hz but corresponding N2 peaks > 12.5 Hz), as is evident from the relatively large dots in this range in Figure 1B. In contrast, slow sigma peaks were often very shallow (small dots) or entirely absent in the channel-averaged spectra, resulting in identified peaks in far fewer subjects (N2: 16, including one subject with peak at 12.5 Hz but corresponding N3 peak < 12.5 Hz; N3: 20). These findings illustrate that two separate sigma peaks can be uncovered from channel-averaged spectra in roughly half the subjects.

Importantly, there was considerable variation between subjects in the precise frequencies of slow (N2: 11.1 ± 1.0 Hz [mean ± SD]; N3: 10.9 ± 0.8 Hz; no stage difference: *t*_(14)_ = 1.2, *P* = 0.24) and fast sigma peaks (N2: 13.5 ± 0.6 Hz; N3: 13.4 ± 0.6 Hz; trending to minimal stage difference: *t*_(23)_ = 1.9, *P* = 0.07), without consistent separation into distinct slow and fast bands across subjects. Importantly, while the demarcation line at 12.5 Hz does a reasonable job for the group, some individuals’ peaks in Figure 1A are centered right on, or very close to, that boundary (e.g., S4, S6, S23 and S25). We also note that many clear slow sigma peaks would have been missed entirely if the lower slow sigma boundaries were set at a more conventional 11 Hz, rather than 9 Hz (e.g., S9, S16, S18, S19). Naturally, this issue would only be exacerbated if this threshold were increased further. Similarly, depending on the precise boundaries used, closely spaced peaks could be lumped together into just one of the spindle classes. Thus, fixed frequency criteria do not adequately capture the natural variation of spindle frequencies across individuals.

As expected, N2 and N3 power profiles were often considerably different within the same individual (Figures 1A,B), most notably in the slow oscillation (0.5–2 Hz) and delta (2–4 Hz) range. Indeed, quantitative analyses indicated that an individual’s N2 and N3 spectra were generally as different as spectra from different individuals (Supplementary Results). N2 and N3 differences in sigma amplitude were also evident. While spectral peak frequencies were relatively consistent for fast spindles, they did not correspond well between N2 and N3 in the slow sigma range (e.g., S3, S8, S18; note the disparity in location between magenta N2 dots and green N3 dots). Indeed, for subjects having identifiable peaks in both stages, peak frequencies were highly correlated across N2 and N3 for fast sigma (*R* = 0.91, *P* < 10^−9^), but less robustly for slow sigma (*R* = 0.53, *P* = 0.04). Thus, selection of slow and fast sigma bands has to address variability both across subjects and between sleep stages.

We also examined power spectra within individuals across the two available recording nights, and confirmed previous evidence that spectral profiles are exceptionally similar across nights (De Gennaro et al., 2005, 2008; Lewandowski et al., 2013), including stable N2-N3 differences (Figure 1C; see Supplementary Figure S1 for spectra of all individuals). Quantitative analyses further supported this notion, including highly accurate cross-night subject identification around 90% (Supplementary Results).

In sum, NREM spectra differ considerably both across individuals and within individuals for N2 and N3, including variation in sigma peak frequencies. Nonetheless, spectral differences are highly stable across nights, suggesting the existence of similarly trait-like sigma peak frequencies. We return to this issue in more detail in the following sections.

#### Single-Channel Spectra

While averaging spectra across channels offers a topographically unbiased perspective, this approach may also obscure and distort spectral peaks present on a limited number of channels. Thus, the appearance of unitary sigma peaks in Figure 1A for some subjects may reflect the merger of closely spaced slow and fast sigma peaks, or the attenuation of slow sigma peaks if they are only present on a few frontal channels.

We therefore turned to examining spectra on individual frontal (Fz) and parietal (Pz) channels where slow and fast spindles are most frequently reported (Tamaki et al., 2008; Ayoub et al., 2013). Figure 2A shows data for subject S10, with clearly separable slow frontal (blue) and fast parietal peaks (red), during both N2 (dashed) and N3 (solid). In a more ambiguous example (S6, Figure 2B), posterior channel Pz exhibited clear peaks around 13 Hz, while frontal channel Fz expressed a peak at 11.5 Hz during N3, presumably reflecting slow spindles. However, Fz displayed a much faster 12.9 Hz peak during N2, ostensibly signaling fast rather than slow spindle activity. In yet another case (S4, Figure 2C), both the frontal and parietal channels showed peaks around 13 Hz with no suggestion whatsoever of a separate slow sigma peak. Applying the same peak detection method as before, single-channel sigma peak isolation was numerically improved relative to the channel-averaged approach for Pz-based fast (N2: 28 vs. 27; N3: 27 vs. 24) and Fz-based slow spindles (N2: 19 vs. 16; N3: 24 vs. 20). Detailed values of single-channel detection success are presented in Table 1. Moreover, peak frequencies were highly correlated across nights (fast N2: *R* = 0.79, *P* < 10^−6^; fast N3: *R* = 0.92, *P* < 10^−11^; slow N2: *R* = 0.81, *P* < 10^−4^; slow N3: *R* = 0.79, *P* < 10^−5^), further affirming the stability of sleep spectral features.

**Table 1:** Sigma peak detection using single-channel and component-based spectra.

		Slow		Fast	
		N2	N3	N2	N3
*Night 1*	*Single-channel*	68%	86%	100%	96%
	*Component*	96%	100%	100%	100%
	*Z*	2.8	2.1	0	1
	*P*	0.005	0.04	1	0.31
*Night 2*	*Single-channel*	79%	93%	100%	100%
	*Component*	93%	93%	100%	93%
	*Z*	1.5	0	0	−1.0
	*P*	0.13	1	1	0.31

*Percentage of subjects (*n* = 28) where sigma peaks could be identified in the slow and fast sigma ranges, for N2 and N3 in both nights. The z statistic was used to compare proportions between the single-channel and component-based approaches*.

However, when we examined the correspondence of sigma peak frequencies across N2 and N3, we obtained disparate results for slow and fast spindle activity. Fast sigma frequencies, averaged across the two nights, were highly correlated across stages (Figure 2D; *R* = 0.92, *P* < 10^−11^), with a small, but significant difference in frequency between N2 and N3 (13.4 ± 0.6 vs. 13.3 ± 0.6 Hz, *t*_(27)_ = 2.3, *P* = 0.03). In contrast, for slow sigma we found no reliable association between stages (*R* = 0.36, *P* = 0.09), and observed a significant difference between N2 and N3 frequency that was much greater than for fast sigma (11.2 ± 1.0 vs. 10.7 ± 0.8 Hz; *t*_(22)_ = 2.2, *P* = 0.04), differing in individual cases by up to 3.3 Hz. The latter result confirms previous observations that peak frequencies of slow sigma can differ substantially between N2 and N3 (Mölle et al., 2011).

In sum, these findings indicate that while a single-channel vs. channel-averaged approach allows sigma peak detection in more individuals, this does not remedy the inconsistencies in slow sigma peak location observed across sleep stages. However, addressing this issue is crucial to accurately distinguish slow from fast spindle activity; we turn to a proposed resolution next.

### Component-Based Analyses

We sought to overcome the limitations of channel-based approaches to determine subject-specific spindle frequencies by harnessing the spatio-spectral structure inherent to multi-channel EEG recordings. Conceptually, the main obstacle to identifying slow sigma peaks is that, for any individual, it is not known *a priori* which channels express slow sigma most clearly and are least affected by interfering fast sigma activity. By determining an optimal combination of channels that enhances slow as opposed to fast sigma activity, and vice versa, the problem of identifying individual subjects’ slow and fast sigma frequencies becomes tractable.

Computationally, we derived the desired channel combinations using a linear spatial filtering approach based on generalized eigendecomposition (GED). These spatial filters were then applied to the original EEG, resulting in “components” that we subsequently analyzed in the frequency domain (see “Materials and Methods” Section). Figures 2E–G show the results of such analyses in the same three subjects used for the single-channel approach. For S10, who already expressed clear slow and fast sigma peaks in single-channel spectra (Figure 2A), the GED-based component approach identified highly similar peaks (Figure 2E). More importantly, and in sharp contrast to the channel-based approach, clear slow peaks were also identified for both S6 and S4 during both N2 and N3 sleep (Figures 2F,G). For S6, where the frontal channel appeared sensitive to fast rather than slow spindle activity during N2 (Figure 2B), components now identified slow peaks in both N2 and N3, with peaks frequencies only 0.6 Hz apart (Figure 2F). Remarkably, whereas S4 showed no inkling of slow sigma activity based on channel Fz during either N2 or N3 (Figure 2C), the decomposition approach identified components in the slow sigma range for both N2 and N3 with highly similar peak frequencies (Figure 2G). Importantly, these slow components were distinguishable from components with peaks in the fast sigma range, suggesting this technique is able to identify closely spaced, but distinct, oscillatory rhythms. Critically, these findings demonstrate that relying on channel-based spectra can lead to erroneous conclusions regarding the frequency, and even existence, of slow spindle activity.

Using this GED component method, we identified fast peaks for a total of 110 of 112 subject-night-sleep stage recordings (all but 2 second-night N3 recordings), similar to channel-based detection (Table 1). In contrast, for slow sigma, component-based peaks could now be isolated for all but three and two subject-night recordings for N2 and N3, respectively. This constitutes a significant increase in the proportion of detected slow sigma peaks for night 1 (Table 1, N2: *P* = 0.005; N3: *P* = 0.04), but not night 2 (N2: *P* = 0.13, N3: *P* = 1). As with channel-based peak detection, cross-night correlations were very high for fast sigma (N2: *R* = 0.93, *P* < 10^−11^; N3: *R* = 0.94, *P* < 10^−11^) and slow sigma peaks (N2: *R* = 0.77, *P* < 10^−5^; N3: *R* = 0.59, *P* = 0.0015), indicating this method identified components with similar spectral properties across nights.

Repeating the cross-stage analyses for component-based sigma frequencies, we found that fast sigma peak frequencies were highly correlated as before (Figure 2H; *R* = 0.95, *P* < 10^−13^), with a small, but significant difference between N2 and N3, similar to the channel-based approach (13.5 ± 0.6 vs. 13.4 ± 0.6 Hz, *t*_(27)_ = 2.7, *P* = 0.01). For slow sigma, however, peaks now also showed a strong correlation between stages (Figure 2H; *R* = 0.68, *P* < 10^−4^; cf. *R* = 0.36, *P* = 0.09 for single-channel analyses, above). Moreover, slow sigma frequency no longer differed significantly between N2 and N3 (11.0 ± 0.8 vs. 10.8 ± 0.7 Hz; *t*_(27)_ = 1, 4, *P* = 0.15). Thus, the GED component approach identified highly similar peaks in N2 and N3, suggesting that the same underlying oscillatory phenomena are present, and may be captured, during both light N2 and deep N3 sleep. We also directly compared sigma frequencies as determined from the single-channel and component approaches and found that peak frequencies are not substantially shifted by the component approach (Supplementary Results).

In total, unambiguous sigma peaks that were stable across N2 and N3 in both nights could be isolated for all 28 subjects for fast spindles, and for all but three individuals for slow spindles. Of note, applying the same strict criteria to channel-based spectra would result in a further loss of nine subjects for slow spindle analyses. Thus, the component-based approach reduced the amount of data excluded from downstream slow spindle analyses by 75%. Mean fast spindle frequency was 13.5 ± 0.6 Hz (range: 12.5–15.4 Hz), while average slow spindle frequency was 10.9 ± 0.7 (range: 9.3–12.0 Hz), well in line with previous studies (Mölle et al., 2011; Ujma et al., 2015). Not surprisingly, a paired *t*-test showed these frequencies to be significantly distinct (*t*_(24)_ = 12.2, *P* < 10^−11^). Similar to previous reports (Kokkinos and Kostopoulos, 2011), we found no reliable correlation between slow and fast spindle frequencies (*R* = −0.30, *P* = 0.15).

In sum, component-based sigma peak frequencies were highly replicable across nights and between sleep stages, for both fast, and crucially, for slow spindles. While single-channel spectra yielded similarly consistent results for fast sigma activity across nights and stages, these correspondences were much poorer for channel-based slow sigma activity. Together, these findings demonstrate that subject-specific spindle frequencies can be identified with higher accuracy using a GED-based spatial filtering approach. Supplementary Matlab code illustrates how to implement the GED-based sigma peak detection approach for several example sleep recordings.

### Topographical Analyses

We now turn to topographical analyses of spindle activity defined by the individualized sigma peaks identified in the previous section. These examinations follow three main threads. First, given the novelty of the GED-based component approach to isolate subject-specific sigma frequencies, these analyses aim to provide crucial validation of our method. In order to demonstrate that the identified oscillatory frequencies correspond to physiological slow and fast spindles, individually targeted spindle activity should replicate key findings regarding slow frontal and fast centro-parietal spindle topography (Werth et al., 1997; Zeitlhofer et al., 1997). However, these previous reports did not statistically compare these spatial patterns, nor have N2 and N3 spindle activity patterns been contrasted directly, motivating more rigorous permutation-based topographical group analyses. Second, taking advantage of the increased sensitivity afforded by our method, we take a detailed look at the spatial properties of sleep spindle activity at the individual level, and find that group effects mask substantial individual variability. Third, we compare spindle topographies between several often-used metrics of spindle activity (sigma power, spindle density, spindle amplitude), and observe that these topographies show only moderate resemblance on the individual level. Combined, these analyses offer several new insights regarding topographical spindle dynamics.

#### Group-Level Slow and Fast Sigma Power Topographies

In order to carry out topographical analyses, we set individualized sigma ranges as a 1.3 Hz band centered on the mean peak frequency across N2 and N3. Due to overlapping slow and fast sigma ranges in two individuals, we included 26/28 individuals for fast spindle analyses and 24/28 for slow spindles (see “Materials and Methods” Section). We then averaged every electrode’s normalized power spectrum across frequency bins corresponding to that individual’s slow and fast sigma range. We performed this procedure separately for N2 and N3, and for each night. In what follows, we report data from night 1, unless otherwise stated.

**Figure 3:**
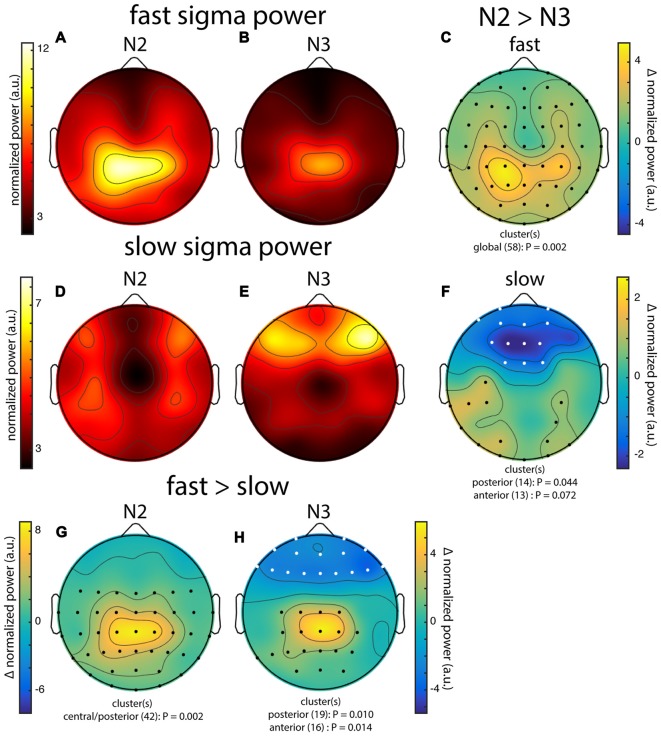
Group topographies of fast and slow sigma power during N2 and N3 from night 1. **(A)** N2 fast spindles. **(B)** N3 fast spindles. **(C)** N2 > N3 fast spindles difference. **(D)** N2 slow spindles. **(E)** N3 slow spindles. **(F)** N2 > N3 slow spindles difference. **(G)** N2 fast > slow difference. **(H)** N3 fast > slow difference. Significant electrodes from paired tests with cluster correction indicated on difference maps as black (positive) and white (negative) dots. Cluster size(s) (number of electrodes) and *P* value(s) indicated below each difference map. Note the different color scales for fast **(A,B)** vs. slow spindles **(D,E)**, as well as different scales for each difference map.

Across subjects, fast sigma power showed a clear centro-parietal topography, both during N2 and N3 (Figures 3A,B). Moreover, performing topographical statistics with cluster correction, we found that fast sigma power was significantly elevated during N2 relative to N3 in one large cluster comprising all electrodes (*P* = 0.002, Figure 3C), although the difference was largest at parietal sensors. In contrast, we saw distinctly different topographical profiles for slow sigma power. While during N3 slow spindles exhibited a clear frontal topography as expected (Figure 3E), slow sigma activity during N2 was expressed in a bilateral fronto-central fashion, albeit with reduced power (Figure 3D). Statistical analyses (Figure 3F) revealed that slow sigma power was higher in N3 than N2 in a frontal cluster comprising 13 electrodes (*P* = 0.07), whereas the reverse was found for a posterior cluster of 14 electrodes (*P* = 0.04). The distinctly low slow sigma power at Fz during N2 is noteworthy, as this electrode is often used to quantify slow spindles (Tamaki et al., 2008; Ayoub et al., 2013).

We also directly contrasted fast and slow spindle activity and observed that, during N2, fast sigma power was significantly enhanced over a wide central-to-posterior region (*P* = 0.002, Figure 3G). In fact, while fast spindles clearly had a parietal focus, frontal fast spindle power during N2 was still numerically greater than slow sigma power. This observation could explain why N2 slow sigma peaks are not readily observed in channel data and may be easily overshadowed by fast sigma peaks. For N3, we similarly observed greater fast vs. slow spindle power over parietal regions (*P* = 0.010, Figure 3H), and additionally, enhanced slow vs. fast power over a frontal cluster (*P* = 0.014, Figure 3H).

These group-level findings, based on our component-based extraction of individualized spindle frequency bands, confirm the differential topographical expression of frontal slow and parietal fast spindles, and N2 vs. N3 differences in fast spindle activity (Werth et al., 1997; Mölle et al., 2011). In addition, we report a more distributed slow spindle topography during N2 that was statistically distinct from the frontal pattern during N3. Thus, while slow spindles are most prominent over frontal areas during periods rich in slow oscillations, they extend to more parietal areas during light sleep. Importantly, we observed highly similar topographies and reproducible significant clusters when we analyzed data from night 2 (not shown). Encouraged that the individualized spatial filtering approach produced expected results, we decided to have a deeper look at the individual topographies contributing to the group effects.

#### Individual Slow and Fast Sigma Power Topographies

**Figure 4:**
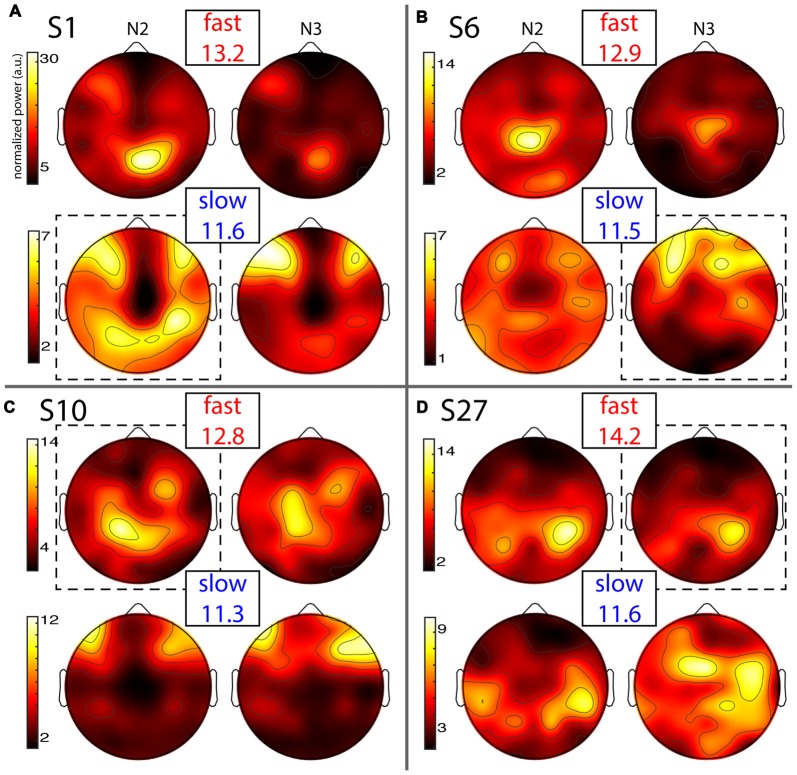
Individual topographies of fast and slow sigma power from night 1. For each subject panel **(A–D)**, the top row displays normalized fast sigma power, the bottom row shows slow sigma power, and the left and right columns show N2 and N3 topographies, respectively. Note the different color scales for fast vs. slow spindles. Numbers below “fast” and “slow” indicate each individual’s peak sigma frequencies in Hz. Dashed boxes indicate topographies used for cross-night comparisons of Figure 5.

Individual topographies of slow and fast sigma power from night 1 are shown for four subjects in Figure 4, including two whose selected components were shown in Figure 2. Inspection of scalp maps revealed both commonalities and considerable variability across subjects.

Fast spindles (top row in every panel) were expressed across the entire cortex, but with typical hotspots over central and parietal areas, although the exact topographies varied substantially. In line with group effects, fast sigma power was distinctly greater in N2 compared to N3 for some subjects (Figures 4A,B), but not others (Figures 4C,D). These findings also match the shape of individual power spectra in Figure 1A. Regarding slow spindle activity, while three subjects exhibited a clear frontal topography during N3 (Figures 4A–C), another had a more distributed, and distinctly non-frontal, pattern during deep sleep (Figure 4D). During N2, slow sigma topographies were even more variable, with one subject having a frontal distribution matching the one seen in N3 (Figure 4C), a second having both frontal and parietal hotspots (Figure 4A), another having just posterior clusters (Figure 4D), and yet another having a rather distributed profile (Figure 4B). We note that the observed individual topographical variability emphasizes the difficulty of detecting slow sigma peaks from a single frontal channel. For example, S1 (Figure 4A) did not express much slow spindle power at channel Fz, and, additionally, had higher fast than slow sigma power even over frontal regions. Indeed, this subject only demonstrated one unitary sigma peak in the power spectrum of Figure 1.

Despite overall power differences, each individual’s N2 and N3 topographies appeared quite similar within a spindle class. In order to quantify this, we correlated N2 and N3 sigma power across all electrodes for each individual, separately for slow and fast spindle classes. Thus, this approach assesses the similarity between topographical N2 and N3 patterns within subjects, irrespective of potential stage-dependent differences in absolute power. We found quite strong correlations of around 0.5 and 0.8 for slow and fast sigma, respectively (Table 2, *N2 vs. N3*). These values were significantly greater than zero both at the group level and for at least 75% of individuals across N2 and N3. However, these results do not indicate whether similar correspondences might be found when correlating N2 and N3 sigma profiles from different subjects. We therefore trained k-nearest neighbor classifiers on individuals’ N2 sigma profiles and tested them on N3 profiles from the same night, and vice versa. Recognition rates were highly significant, at approximately 45% and 75% for slow and fast sigma (Table 2, *N2 vs. N3*). Thus, these findings suggest the same underlying subject-specific spindle generators are active across light N2 and deep N3 sleep states, most clearly for fast spindles.

**Table 2:** Within-subject similarity of sigma power topographies across sleep stages and spindle classes.

Comparison				Correlation		Classification	
			*N*	*R*	*P*_corr_ < 0.05	I ≥ II	II ≥ I
*N2 vs. N3*	*Night 1*	*Slow*	24	0.46 ± 0.37***	75%	42%***	46%***
		*Fast*	26	0.78 ± 0.21***	96%	77%***	69%***
*Slow vs. Fast sigma*	*Night 1*	*N2*	24	0.33 ± 0.35***	67%	25%***	38%***
		*N3*	24	0.19 ± 0.35*	63%	33%***	46%***

R: Pearson correlation coefficient (mean ± SD, across individuals); P_corr_ < 0.05: percentage of subjects with False Discovery Rate-corrected *P* values < 0.05; I ≥ II and II ≥ I: classification direction. Significance levels (correlation: one-sample *t*-test vs. zero; classification: binomial test) indicated by * < 0.05; *** < 0.001.

As evident from Figure 4, each individual’s slow and fast sigma topographies appeared to correspond poorly. Quantifying the degree of similarity between these spatial profiles, we found that slow and fast sigma topographies had average correlation coefficients of 0.3 and 0.2 during N2 and N3, respectively, indicating only limited within-subject correspondence of slow and fast sigma expression across the scalp (Table 2, *slow vs. fast sigma*). Again, however, these results do not account for the baseline similarity of slow and fast sigma profiles from different subjects. Using classifiers, we asked if knowing individuals’ slow sigma profiles would allow us to recognize them from their fast sigma topography, and vice versa. Across N2 and N3, we obtained significantly above chance cross-spindle-type classification rates of around 30% when trained on slow sigma and tested on fast sigma, and of around 40% when trained on fast sigma and tested on slow (Table 2, *slow vs. fast sigma*). These findings indicate that, even though an individual’s spatial profiles of slow and fast sigma expression are quite distinct (low correlation), they still share a sufficient degree of commonality to allow differentiation from other individuals’ sigma topographies in at least a third of our subjects. Overall, however, individual slow and fast sigma profiles were much less similar than N2 and N3 topographies of the same spindle type, suggesting these spindle classes are largely distinct.

**Figure 5:**
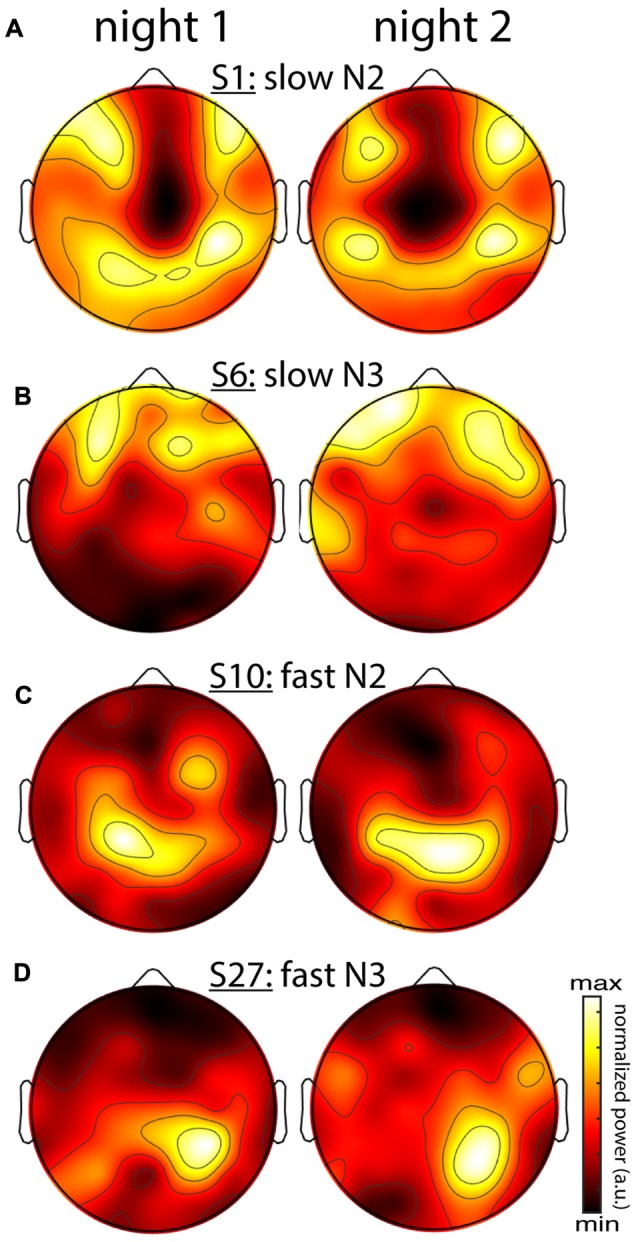
Stability of individual differences in sigma power topographies across nights. **(A)** Slow N2 sigma for subject S1. **(B)** Slow N3 sigma for subject S6. **(C)** Fast N2 sigma for subject S10. **(D)** Fast N3 sigma for subject S27. To emphasize topographical similarity, color scales were scaled between minimum and maximum value for each topography.

So far, it is unclear whether individual differences in topographical expression of sigma power constitute stable traits, or rather, reflect night-specific differences in brain state that may be equally pronounced for the same individual across nights. As shown for the examples in Figure 5, however, individual spatial differences were highly stable across nights, for both slow and fast sigma power, and for both N2 and N3. To quantify this effect, we correlated sigma power across all electrodes between the two nights for each individual. We found substantial evidence for individual topographical stability across nights, with correlation values of around 0.6 for both slow and fast sigma topographies, and during both N2 and N3 (Table 3, *sigma power*). Note how these cross-night correlations were much higher than cross-spindle-type correlations within the same night. We again turned to classifiers to ask if individuals can be recognized across nights based on their spatial expression of sigma activity. Recognition performance was significantly above chance for all analyses, but successful in up to three times as many individuals for N2 than N3, and higher for slow than fast sigma profiles (Table 3, *sigma power*). Whereas cross-night N3 fast sigma topographies were only slightly more similar within than between individuals, leading to classification rates around 20%, this difference was much greater for N2 slow sigma profiles, yielding recognition accuracy around 65%. The latter observation is noteworthy given that slow sigma peaks were most difficult to isolate from channel-based spectra in this sleep stage, indicating that meaningful slow sigma topographies may be extracted even under taxing circumstances.

**Table 3:** Within-subject similarity of topographies across nights.

Comparison				Correlation		Classification	
			*N*	*R*	*P*_corr_ < 0.05	I ≥ II	II ≥ I
*Sigma power*	*Slow*	*N2*	24	0.67 ± 0.21***	96%	71%***	58%***
		*N3*	24	0.56 ± 0.25***	92%	38%***	46%***
	*Fast*	*N2*	26	0.69 ± 0.20***	96%	46%***	54%***
		*N3*	26	0.53 ± 0.23***	88%	23%***	15%**
*Spindle density*	*Slow*	*N2*	24	0.60 ± 0.26***	92%	33%***	46%***
		*N3*	24	0.52 ± 0.32***	75%	25%***	46%***
	*Fast*	*N2*	26	0.46 ± 0.22***	77%	35%***	23%***
		*N3*	26	0.24 ± 0.25 ***	46%	15%**	19%***
*Spindle amplitude*	*Slow*	*N2*	23	0.64 ± 0.25***	91%	43%***	48%***
		*N3*	17	0.57 ± 0.26***	88%	47%***	47%***
	*Fast*	*N2*	25	0.59 ± 0.26***	88%	48%***	40%***
		*N3*	16	0.53 ± 0.22***	88%	31%***	38%***

R: Pearson correlation coefficient; P_corr_ < 0.05: percentage of subjects with False Discovery Rate-corrected *P* values < 0.05; I ≥ II and II ≥ I: classification direction. Significance levels (correlation: one-sample *t*-test vs. zero; classification: binomial test) indicated by ** < 0.01; *** < 0.001. Note: cases where spindles were not detected on every channel were excluded from spindle amplitude analyses.

For all preceding and forthcoming classification analyses, it should be mentioned that recognition scores are influenced by both individual stability and between-subject variability. Higher “baseline” topographical similarity between different individuals for a particular sleep stage or spindle class could make it more difficult to differentiate between individuals, even if within-subject topographies are highly similar. Thus, the classifier approach takes between-subject variability into account, complementing the topographical correlation analyses that do not.

Together, these subject-specific analyses demonstrate that individual patterns of sigma expression are relatively stable across sleep states, but much less so across slow and fast sigma bands, providing additional support for the existence of two distinct spindle classes. Moreover, individual variability in topographical sigma expression is stable across nights, most prominently for N2 sleep. As such, individual topographical variability of both slow and fast spindles appears to reflect another individual trait of NREM sleep, similar to the cross-night stability of power spectra (Supplementary Results). In sum, while the previous section demonstrated clear indications of canonical slow and fast sigma topographies across subjects, these group effects mask substantial individual variability that must be taken into consideration when analyzing the spatial properties of spindle activity.

#### Topographies of Individually Detected Spindles across and within Subjects

A potential concern from the previous analyses is that by focusing on sigma power we do not treat spindles as distinct events with designated starts and ends. More generally, it is often assumed, at least implicitly, that sigma power and properties of individually detected spindles (e.g., spindle density, spindle amplitude) capture largely similar aspects of underlying spindle activity. However, as we are unaware of studies directly comparing these metrics, we next turn to examinations of group-level and subject-specific topographies of discrete spindle properties, and relate these to the sigma power patterns described in the previous sections.

We used an automated spindle detector (Cox et al., 2012, 2014a) to isolate individual sleep spindles using the subject-specific frequencies identified earlier. Briefly, the algorithm filters Laplacian-transformed channel data in the individualized sigma range of interest, and applies upper and lower thresholds based on characteristics of the N2 sigma envelope (see “Materials and Methods” Section). Of note, this threshold approach dynamically adapts to the level of sigma signal present at each channel, thus contrasting with the sigma power approach that expressly does not account for such differences. Moreover, by running the algorithm twice, targeting slow and fast sigma ranges separately, different thresholds are applied for slow and fast spindle detection, aiding in isolating different-amplitude spindles. For example, upper thresholds were significantly higher across subjects for slow vs. fast spindle detection on channel Fz (0.20 ± 0.06 vs. 0.18 ± 0.07 μV/cm^2^, *t*_(27)_ = 3.6, *P* = 0.001), but this relation was reversed on channel Pz (0.17 ± 0.07 vs. 0.21 ± 0.10 μV/cm^2^; *t*_(27)_ = −2.4, *P* = 0.02). After detecting spindle events in this manner, we calculated spindle density (number per minute) and mean spindle peak amplitude for each channel and individual, separately for N2 and N3, and separately for slow and fast spindles.

**Figure 6:**
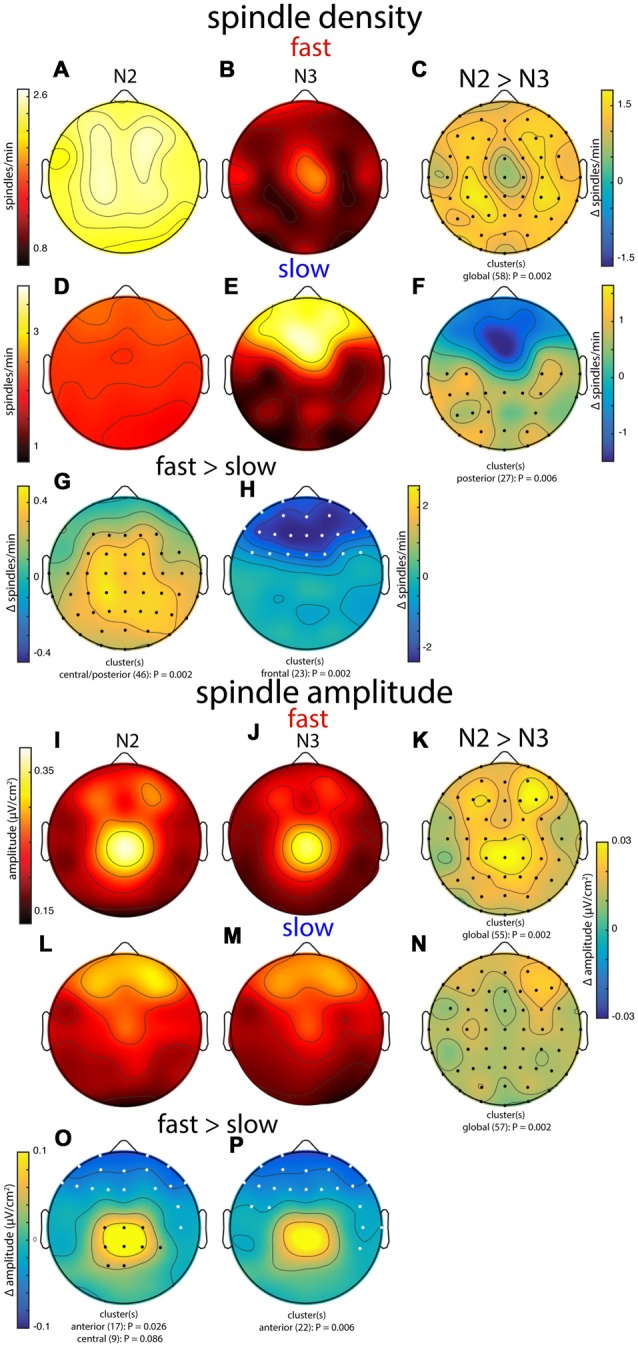
Group topographies of fast and slow spindle density **(A–H)** and spindle amplitude **(I–P)** during N2 and N3. **(A,I)** N2 fast spindles. **(B,J)** N3 fast spindles. **(C,K)** N2 > N3 fast spindles difference. **(D,L)** N2 slow spindles. **(E,M)** N3 slow spindles. **(F,N)** N2 > N3 slow spindles difference. **(G,O)** N2 fast > slow difference. **(H,P)** N3 fast > slow difference. Significant electrodes from paired tests with cluster correction indicated on difference maps as black (positive) and white (negative) dots. Cluster size(s) (number of electrodes) and *P* value(s) indicated below each difference map. Color scales are shared for **(A,B)**, **(D,E)**, **(I,J,L,M)**, **(K,N)**, and **(O,P)**.

In general, group topographies of spindle density and peak spindle amplitude were consistent with sigma power profiles presented in Figure 3. Compared to sigma power, spindle density profiles generally had a more diffuse appearance (Figures 6A,B,D,E), but topographical statistics indicated N2 vs. N3 differences for both fast (Figure 6C) and slow spindles (Figure 6F) similar to those seen for sigma power. Similarly, slow vs. fast spindle density topographies matched those observed for sigma power (Figures 6G,H; noting that different thresholds were applied for detecting these two spindle types). Spindle amplitude topographies also looked similar to sigma power patterns, albeit with a more focal appearance (Figures 6I,J,L,M). Similar to the other metrics, fast spindle amplitude was greater in N2 than N3 (Figure 6K). In addition, slow spindles were also of significantly higher amplitude in N2 vs. N3 (Figure 6N), including frontal regions where values were substantially lower in N2 for sigma power (significantly) and spindle density (numerically). Finally, fast and slow spindle amplitude topographies differed significantly as the other metrics did (Figures 6O,P).

Combined, these group-level topographies provide some insights regarding which aspects of spindle activity contribute to observed sigma power. In particular, the N2 fast sigma power peak over centro-parietal regions (Figure 3) appears to be predominantly driven by enhanced spindle amplitude in this region, but not by spindle density, which was quite uniform across the scalp. In contrast, the bilateral fronto-central profile of N2 slow sigma seems inexplicable in terms of either spindle density or amplitude. Interestingly, both density and amplitude of spindles appear to contribute to the patterns of both fast and slow sigma power in N3.

To examine these group-level observations in more detail, we turned to spatial maps of spindle properties from individual subjects. Similar to sigma power, individual subjects’ spindle density and spindle amplitude profiles were quite variable, and these, too, were generally stable across nights (Table 3, *spindle density* and *spindle amplitude*). However, direct comparisons of cross-night topographical similarity between metrics indicated that spindle density topographies were significantly less stable across nights than sigma power profiles for fast spindles (N2: *t*_(25)_ = 6.0, *P* < 10^−5^; N3: *t*_(25)_ = 6.5, *P* < 10^−6^), but not for slow spindles (both *P* > 0.18). Similarly, spindle density patterns were less stable than spindle amplitude patterns for fast (N2: *t*_(24)_ = 3.4, *P* = 0.002; N3: *t*_(15)_ = 3.8, *P* = 0.002), but not slow spindles (both *P* > 0.30). Complementing this view, we found cross-night subject classification based on spindle density (mean across spindle class, sleep stage, and classification direction: 30 ± 12%) to be significantly lower than sigma power-based (44 ± 18%; *t*_(7)_ = 2.7, *P* = 0.03) and spindle amplitude-based recognition (43 ± 6%; *t*_(7)_ = 4.6, *P* = 0.002). We also observed sigma power profiles to be more similar across nights than spindle amplitude profiles for fast N2 spindles (*t*_(24)_ = 3.0, *P* = 0.006), but not for fast N3 spindles or slow spindles (all *P* > 0.30). However, cross-night classification rates did not differ between sigma power and spindle amplitude (*P* > 0.8). Overall, these findings establish that topographical patterns of sigma power and spindle amplitude are more reliable across nights than spindle density distributions, particularly for fast spindles.

**Figure 7:**
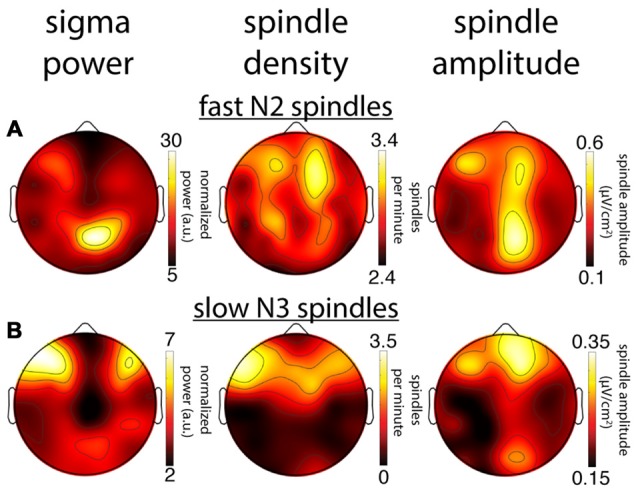
Comparison of topographical maps based on different spindle activity measures for a single subject (S1). **(A)** Topographies of sigma power, spindle density and spindle amplitude for fast spindles during N2. **(B)** Topographies for slow spindles during N3. While this subject’s topographies based on different measures show a reasonable correspondence for slow N3 spindles, spatial agreement is quite poor for fast N2 spindles.

We next compared within-subject topographical patterns of all three metrics from the same night to determine how well they correspond with one another. For illustration, Figure 7 shows topographies from one subject for all metrics, for both fast N2 spindles (Figure 7A) and slow N3 spindles (Figure 7B). In line with the group maps, this subject’s N2 fast sigma power distribution appears to be more strongly driven by spindle amplitude, and to a lesser extent, if at all, by spindle density (Figure 7A). In contrast, the slow N3 power topography appears similarly related to spindle amplitude and spindle density profiles (Figure 7B). These observations suggest that amplitude and prevalence of spindles may contribute to observed sigma power in different ways depending on spindle class and sleep stage.

To quantify these visual observations, we directly compared topographical patterns between each pair of these three metrics within individuals. Detailed values and statistics can be found in Table 4. Overall, within-subject spatial maps based on different metrics showed moderate correspondence, although results depended importantly on sleep stage, spindle class, and which metrics were being compared. Confirming the observations from Figures 3, 5, 7, fast sigma power topographies were more closely related to spindle amplitude than spindle density for both N2 (*t*_(25)_ = 3.3, *P* = 0.003) and N3 (*t*_(17)_ = 2.9, *P* = 0.01). In contrast, slow sigma power topographies were equally poorly related to spindle amplitude and density during N2 (*t*_(23)_ = −0.1, *P* = 0.95), but more similar to spindle density than spindle amplitude topographies during N3 (*t*_(18)_ = −2.0, *P* = 0.06). In terms of subject discriminability, cross-metric subject recognition rates typically did not exceed 40%, except for N3 sigma power vs. spindle density topographies. To place these findings in context, we achieved higher recognition performance for the majority of cross-sleep stage and cross-night comparisons based on the same sigma power metric (Tables 2, 3, Figures 4, 5), than we did for cross-metric comparisons within the same night and sleep stage.

**Table 4:** Within-subject similarity of topographies based on different measures of spindle activity.

Comparison				Correlation		Classification	
			*N*	*R*	*P*_corr_ < 0.05	I ≥ II	II ≥ I
*Sigma power vs. Spindle density*	Slow	N2	24	0.17 ± 0.38*	46%	17%**	13%*
		N3	24	0.62 ± 0.23***	88%	58%***	75%***
	Fast	N2	26	0.24 ± 0.26***	42%	19%***	27%***
		N3	26	0.41 ± 0.23***	73%	54%***	50%***
*Sigma power vs. Spindle amplitude*	Slow	N2	24	0.16 ± 0.32*	38%	21%***	17%**
		N3	24	0.46 ± 0.26***	71%	13%*	21%***
	Fast	N2	19	0.44 ± 0.23***	77%	42%***	31%***
		N3	18	0.56 ± 0.22***	54%	35%***	35%***
*Spindle density vs. Spindle amplitude*	Slow	N2	24	0.38 ± 0.37***	71%	25%***	13%*
		N3	19	0.42 ± 0.36***	63%	21%***	29%***
	Fast	N2	26	0.47 ± 0.20***	84%	42%***	35%***
		N3	18	0.27 ± 0.25***	38%	15%**	19%***

R: Pearson correlation coefficient; P_corr_ < 0.05: percentage of subjects with False Discovery Rate-corrected *P* values < 0.05; I ≥ II and II ≥ I: classification direction. Significance levels (correlation: one-sample *t*-test vs. zero; classification: binomial test) indicated by * < 0.05; ** < 0.01; *** < 0.001. Note: cases where spindles were not detected on every channel were excluded from spindle amplitude analyses.

Overall, these spindle metric comparisons lead to four conclusions. First, sigma power and spindle amplitude maps are more stable across nights than spindle density profiles. Second, spindle amplitude and spindle density contribute differently to sigma power, depending on sleep stage and spindle class under consideration. Third, topographical maps based on different metrics are generally quite distinct even for the same individual. This somewhat unexpected finding suggests that the choice of spindle metric could have important downstream ramifications regarding topographical group maps, statistics, and interpretations. Finally, these findings again show that group-level maps obscure substantial individual topographical variability of spindle expression.

## Discussion

The current study characterized the large between-subject variability of spindle frequencies and topographies, and assessed the correspondence of spatial spindle expression between slow and fast spindle classes, sleep stages, nights, and several metrics of spindle activity. Employing a novel spatial filtering approach to isolate subject-specific frequencies of slow and fast spindle activity, we replicated topographical properties of spindle expression at the group-level (Werth et al., 1997; Zeitlhofer et al., 1997), strongly suggesting that we successfully isolated the oscillatory phenomena of interest. By then taking a more detailed look at topographical aspects of these oscillatory dynamics, and, furthermore, by extending the subject-specific approach from the spectral to the spatial dimension, we made several novel observations about the organization of NREM spindle dynamics.

### Individual Differences in NREM Spectra and Sigma Frequencies

We observed marked individual differences in the shape of NREM power spectra (Figure 1). Analyses across nights confirmed several previous studies demonstrating the existence of robust spectral power fingerprints during sleep (De Gennaro et al., 2005; Lewandowski et al., 2013). Indeed, these profiles have been shown to be the highly heritable (De Gennaro et al., 2008) and remain stable throughout development (Tarokh et al., 2011). In sharp contrast, we observed that N2 and N3 spectral profiles for the same individual show considerable differences within the same night. While spectral differences between N2 and N3 are inevitable given that these stages are defined by the amount of slow wave (<4 Hz) activity, it is perhaps surprising that N2 and N3 spectral profiles are so different in higher frequency bands.

Indeed, one of the most prominent differences between N2 and N3 spectra appeared in the sigma range, and these sigma differences, too, were highly stable across nights (Figures 1A,C). While fast sigma peaks were more pronounced in N2 than N3 across most individuals, slow sigma peaks did not show consistent amplitude differences between N2 and N3. Yet, both individual sigma peak frequencies and individual topographical patterns of sigma power were highly stable across N2 and N3, suggesting that while the broader oscillatory context may change markedly from light to deep sleep, underlying thalamic spindle generators and their influence on neocortex remain relatively fixed. Thus, the extent to which N2 and N3 are seen to be similar depends on which physiological aspects one considers.

The most striking observation from the spectral profiles, however, was the large between-subject variability of sigma range frequencies, and the difficulty this poses for defining slow and fast sigma ranges that can provide adequate group-level separation of fast and slow spindles. Component-based slow and fast sigma frequencies ranged from 9.3 Hz to 12.0 Hz for slow spindles and from 12.5 Hz to 15.4 Hz for fast spindles, similar to what has been described previously (De Gennaro et al., 2005; Ujma et al., 2015). For this reason, it seems any attempt to define spindle activity in terms of fixed spectral criteria is destined to fail in at least some instances. Thus, rather than arguing in favor of specific spectral definitions for slow and fast spindle classes, we believe this variability provides motivation to identify slow and fast sigma peaks in a subject-specific manner (Gottselig et al., 2002; Bódizs et al., 2009; Adamczyk et al., 2015; Ujma et al., 2015). However, we demonstrated that both channel-averaged spectra and spectra derived from single frontal and parietal channels are inadequate in this regard, and in many instances do not lead to reliable estimates of subject-specific sigma frequencies.

### Separating Slow and Fast Sigma Peaks via Spatial Filters

To improve upon channel-based approaches, we employed a novel data-driven technique to identify individualized slow and fast sigma peaks. By making use of the fact that multi-channel EEG data has a spectral structure that is typically correlated between nearby channels, we constructed spatial filters that maximally enhance spectral content in one frequency range at the expense of another. When we compared component-derived spectral peaks to both channel-averaged and single-channel spectra, we found a clear advantage for the spatial filtering approach. Compared to peaks isolated from individual channels, the component-based approach allowed detection of slow sigma peaks in approximately three-quarters of the sample that would have otherwise been lost for downstream analyses. Nonetheless, it is important to note that channel- and component-based sigma peak frequencies were significantly correlated (Supplementary Results), reflecting the obvious dependence of GED-derived spindle peaks on individual channel information.

Several channel-based approaches have been proposed to identify subject-specific sigma ranges (Bódizs et al., 2009; Mölle et al., 2011; Ujma et al., 2015). While we did not directly compare our technique to these approaches, we see important benefits to the GED-based spatial filtering approach. First, because it is data-driven, it makes no *a priori* assumptions regarding which channels exhibit slow or fast spindle activity. Indeed, individual topographical maps exhibited substantial variability in local spindle expression, such that anterior vs. posterior channel selection may not always successfully resolve distinct peaks (Figure 4D). Second, our approach consistently returned highly similar slow sigma frequencies for N2 and N3. This contrasts with observations of less well-matched slow sigma peaks in N2 and N3 when using spectra averaged across groups of channels (Mölle et al., 2011), or single-channel spectra (this study). Likely, this poor correspondence is due to fast sigma peaks overshadowing slow sigma peaks during N2 and thereby shifting peak locations, an issue that the GED approach inherently tries to avoid by increasing spectral power in one sigma band relative to the other. Several methodological considerations regarding the dependence of the GED approach on the Laplacian transform, initial filter settings, and number of required channels, are discussed in the Supplementary Discussion. Taken together, we suggest our method is a more sensitive technique to robustly identify individuals’ fast and especially slow spindle frequencies than those previously described. Based on this validation of the GED-based approach, we analyzed the spatial characteristics of sleep spindles.

### Sleep Spindle Topographies

We analyzed topographical patterns of spindle expression both at the group and individual level. Group-wise, we observed typical differential topographies of slow and fast spindles, suggesting we succeeded in separating the two spindle classes. Canonical slow-frontal vs. fast-central topographies were observed in N3 for all spindle metrics (Figures 3, 6). In N2, we also observed a central expression of fast spindle activity for sigma power and spindle amplitude, but a less typical fronto-central topography for slow sigma power, and a relatively uniform distribution of both slow and fast spindle densities. Direct statistical comparisons of slow and fast spindles supported distinct topographies for most sleep stage/spindle class/spindle metric combinations. We also observed stronger fast spindle activity during N2 than N3, regardless of metric, consistent with earlier observations (Werth et al., 1997). In contrast, frontal slow sigma power and slow spindle density were greater during N3 than N2, consistent with more pronounced slow sigma spectral peaks in N3 than N2. Here, it should be noted that N2 slow spindles have not been examined in detail previously, likely because channel-based spectra did not unambiguously indicate their presence during N2. Thus, the increased sensitivity afforded by targeting subject-specific spindle frequencies uncovers subtle differences in sigma power expression during N2 and N3 sleep that might otherwise be overlooked.

When we examined in detail individual topographical profiles of spindle expression, we observed substantial individual differences (Figure 4), refining earlier findings (Werth et al., 1997; Zeitlhofer et al., 1997; Finelli et al., 2001). Importantly, individual topographical variability was stable both across sleep stages and nights (Figure 5), indicating these patterns constitute robust traits. Together, these findings underscore that group averages reflect a mixture of quite idiosyncratic spatial spindle patterns.

What could be the cause of such marked individual topographical variability? First, it is possible that spatial variability is due to individual differences in cortical folding patterns, affecting how intracortical spindle signals summate and propagate to the scalp. Second, different individuals might have different anatomical or functional thalamocortical wiring profiles, resulting in different spatial profiles of spindle expression. Indeed, between-subject variability in white matter tracts, including fibers intrinsic to and surrounding the thalamus, is known to be related to spindle power and density (Piantoni et al., 2013). More generally, there is substantial evidence for the existence of localized cortical and subcortical spindles (Nir et al., 2011; Frauscher et al., 2015; Kim et al., 2015; Piantoni et al., 2016) and the potential role of localized spindles in processing specific memories (Clemens et al., 2005, 2006; Nishida and Walker, 2007; Bang et al., 2014; Cox et al., 2014a). Thus, an intriguing possibility is that individual differences in topographical spindle expression have functional implications and underlie individual differences in cognitive processing.

Regardless of the question of function, topographical heterogeneity may be an undesirable source of variance in research designs choosing a specific channel for spindle analysis. For example, ranking subjects according to sigma power on channel Cz will, in all likelihood, yield a different order from one based on each individual’s channel of maximum sigma power. As a result, differences between experimental or clinical groups could stem from these groups having different cortical regions with maximal spindle activity, either systematically or by chance. Similarly, experimental studies testing topographical hypotheses might benefit from taking spatial variability into consideration by recording a baseline night prior to experimental manipulation.

### Not All Spindle Metrics Are Alike

Beyond differences in spectral definition, studies of spindles report a host of different metrics intended to capture (specific aspects of) underlying spindle activity. Classical measures of sigma power are reported along with various properties derived from individually detected spindles (e.g., spindle density, amplitude, peak frequency, power, duration, integral, etc.). While it is well understood that different metrics are, in a trivial sense, distinct, they often appear to be lumped together when relating findings from different studies.

When we compared individuals’ spatial topographies between different spindle activity metrics and across nights, we made several observations. First, amplitude-related topographies (sigma power and spindle amplitude) were more stable across nights than spindle density patterns. This observation matches a report evaluating the stability of spindle characteristics derived from a single channel (Eggert et al., 2015). Second, spindle amplitude and spindle density were found to contribute differently to sigma power, depending on sleep stage and spindle class under consideration. Whereas fast sigma power was more strongly driven by spindle amplitude than spindle density across sleep stages, the opposite was found for slow sigma during N3. Third, while individuals’ topographical patterns of sigma power, spindle density, and spindle amplitude showed above-chance levels of correspondence, cross-metric subject classification was rather low. In general, neither spindle density nor spindle amplitude appeared greatest where sigma power was highest. Similarly, sites where spindles were more plentiful did not necessarily have larger spindles.

How do these various metrics relate? Sigma band power, as a relatively crude measure of spindle activity, likely includes noise unrelated to physiological events of interest, but also captures meaningful activity of low amplitude and short duration spindles that do not register with specialized spindle detection algorithms. Conversely, spindle detection methods are forced to make ultimately arbitrary decisions regarding amplitude and duration criteria, potentially leading to non-detection of relevant spindle activity (for the difficulties associated with validating automated spindle detectors against human scorers, see Warby et al., 2014; O’Reilly and Nielsen, 2015). Anecdotally, we often observed in the raw EEG trace brief, but prominent, increases in sigma amplitude during slow oscillation up states that ended abruptly with the onset of subsequent down states. In many cases, however, these sigma events were shorter than the 400 ms criterion employed in our current algorithm and were not counted as spindles. In the absence of ground truth, we have no clear preference for taking a sigma power or spindle detection approach, nor do we advocate specific spindle detection criteria or particular spindle metrics. From a practical standpoint, however, the greater test-retest reliability of amplitude-based spindle measures may provide motivation to prefer these indices to the less reliable spindle density estimates. Regardless, it is important to realize that different metrics capture different components of spindle activity and cannot be assumed to correspond to one another in a straightforward fashion.

### On the Existence of Slow and Fast Spindles

While the evidence for distinct slow and fast spindles is surprisingly equivocal in the animal literature (Terrier and Gottesmann, 1978; Schwierin et al., 1999; Kim et al., 2015), our findings are well in line with the current notion of two distinct types of human spindles (Schabus et al., 2007; Mölle et al., 2011; Ayoub et al., 2013; Cox et al., 2014b; Staresina et al., 2015; Klinzing et al., 2016; Purcell et al., 2017). Indeed, our group-level topographical maps agree with previous reports (Werth et al., 1997; Zeitlhofer et al., 1997). More fundamentally, we were able to identify separate, and typically quite narrow, slow and fast spectral peaks using the GED spatial filter approach for every individual (although we did exclude three individuals with inconsistent N2 and N3 slow peaks). It is worth emphasizing that the GED spatial filter approach does not systematically introduce artifactual peaks, as there were still some instances where we could not identify sigma peaks from component spectra. We further verified this notion by performing control analyses in which we applied GED to covariance matrices derived from data filtered in the 16–20 Hz and 20–24 Hz ranges. However, the returned components generally did not contain any peaks at all, or returned a peak that was absent in all other night-sleep stage combinations. This contrasts markedly with the clear congruence of component peak locations between nights and sleep stages in the sigma band.

Previous studies described the existence of an anterior-posterior gradient of spindle frequency (Peter-Derex et al., 2012; Frauscher et al., 2015), but it is unclear how the distinct spectral peaks found here could have emerged from a more continuous distribution of sleep spindle frequencies. Instead, such findings could be equally explained in the framework of a bimodal distribution of slow and fast spindles. As others (Werth et al., 1997; Zeitlhofer et al., 1997; Klinzing et al., 2016) and we have shown, both slow and fast spindles occur widely across the brain, but with more anterior or posterior biases. Thus, averaging over many spindles (or, regarding sigma power, many spectra derived from short data segments) can give rise to a topographical frequency gradient by sampling different proportions of slow and fast spindles at different cortical sites. This issue could be exacerbated further when observations from individuals with different slow and fast spindle frequencies are aggregated.

Similarly, findings of varying spindle frequencies throughout the night or within sleep cycles (Werth et al., 1997; Himanen et al., 2002; Andrillon et al., 2011), may be explained by shifting contributions of slow and fast spindle activity as opposed to the occurrence of progressively slower or faster spindles. Indeed, while GED-based sigma frequencies were stable from N2 to N3 (Figure 2B), channel-based frequencies were much more variable between sleep stages (Figure 2F), indicating that the same underlying combination of slow and fast sigma generators can manifest in the power spectrum as peaks with different locations. While we note that individual spindles tend to decelerate over their course (Andrillon et al., 2011; O’Reilly and Nielsen, 2014; Zerouali et al., 2014), we believe that the current evidence, including our findings, is most consistent with two fundamentally distinct human spindle classes. How this suggestion, based on macroscopic and predominantly non-invasive evidence, relates to underlying thalamocortical circuitry is an open question.

As the 9–12.5 Hz range overlaps substantially with the canonical waking alpha (8–12 Hz) band, it may be argued that our slow sigma range merely captures wake-like alpha intrusions. However, the observed group-level frontal (N3) and fronto-central (N2) topographies were decidedly different from classical occipital alpha distributions, arguing against this interpretation. Previous evidence has also indicated differences between individuals’ waking alpha and slow spindle frequencies (Kokkinos and Kostopoulos, 2011). Finally, we examined phase-amplitude coupling between ~1 Hz slow oscillations and slow and fast spindles, using the same underlying data and the individualized sigma frequencies identified here. These analyses, to be reported in a separate article, revealed expected differential phase preferences of slow and fast spindles, with fast spindles occurring preferentially towards the up state, and slow spindles in the down state (Mölle et al., 2011; Klinzing et al., 2016). Together, these considerations strongly suggest that NREM spectral peaks in the 9–12.5 Hz range do not constitute wake-like alpha activity.

### Practical Recommendations

We offer several suggestions regarding the examination of sleep spindles measured with EEG. First, we believe the field would benefit from targeting subject-specific frequencies. While it was outside the scope of the current work to directly compare individualized spectral bands to a fixed frequency approach (see Ujma et al., 2015 for a comparison), we hope the variability showcased in Figure 1 makes the point. Individuals with highest sigma power levels based on fixed spectral criteria are not necessarily the ones with greatest power when using individually defined sigma frequencies. Fixed frequency criteria could therefore have undesired effects, e.g., when assessing the link between spindle activity and memory. As another concern, even small changes in sigma range settings can have comparatively large effects on an individual’s topography, as “true” slow and fast topographies are differentially enhanced, attenuated, and mixed. While we hope our spatial filtering approach will prove useful in this regard, the precise method employed is less important than adopting an individualized approach in the first place.

Second, when subject-specific frequency selection is not feasible, we suggest fixed criteria be based on visual inspection of power spectra. In our sample, approximate slow and fast spindle ranges of 9–12.5 Hz and 12.5–16 Hz would likely have worked reasonably well. Moreover, based on visual inspection of the distribution of peak frequencies in a sample of 161 individuals (Ujma et al., 2015; their Figure 2) these ranges appear to be a sensible choice for young, healthy individuals. However, we caution against blindly following this suggestion, as slow and fast sigma peak distributions may overlap (Ujma et al., 2015), or may be clustered in different spectral bands in different age ranges or clinical groups (Shinomiya et al., 1999). It is also possible that no sensible demarcation frequency can be found, in which case it may be preferable to not further differentiate spindles into slow and fast classes.

Finally, we recommend careful examination of spindle detection criteria used in the literature. Study conclusions regarding the specific involvement of slow or fast spindles that appear at odds may no longer be when one considers in detail the spectral criteria employed. Specifically, we suggest that studies placing lower bounds for slow spindles at 11 or 12 Hz may have missed their slow spindle target in some subjects, and/or confused fast for slow spindles by artificially separating the fast sigma range into two. Rather than criticizing these studies, however, we simply wish to alert researchers and clinicians that seemingly minor methodological details could have a major impact on the interpretation of results.

## Conclusion

Despite decades of progress, the functional role of sleep spindles in general, and of slow and fast spindles in particular, is still unclear. While some recent evidence suggests fast spindles are more strongly implicated in cognitive processes (Tamaki et al., 2008, 2009; Barakat et al., 2011; Cox et al., 2014a; Rihm et al., 2014; Fang et al., 2017), only few studies finding such links target the purported brain rhythms in an individualized manner (Mölle et al., 2011). But if slow and fast spindles do indeed turn out to serve different functions, to serve the same function differently, or to be differentially affected in neuropsychiatric disorders, it becomes critical that these spindle types be adequately separated. We hope our approach will prove useful in this respect, and we suggest that proper understanding of sleep spindle dynamics and their functional role requires addressing individual variability as much as accounting for shared organizational principles.

## Author Contributions

RC, RS and DSM: conceived and designed the experiments. RC: analyzed the data, contributed reagents/materials/analysis tools, and prepared figures. RC, ACS, DSM and RS: interpreted results and contributed to the writing of the manuscript.

## Conflict of Interest Statement

The authors declare that the research was conducted in the absence of any commercial or financial relationships that could be construed as a potential conflict of interest.

## Supplementary Material

Supplementary code for slow/fast sigma peak separation can be found at https://doi.org/10.6084/m9.figshare.4905677
