# Supplementary material for: Individual Differences in Frequency and Topography of Slow and Fast Sleep Spindles
Source: Front Hum Neurosci. 2017 Sep 5;11:433. doi: 10.3389/fnhum.2017.00433 (PMC5591792; doi:10.3389/fnhum.2017.00433)
Supplement: Supplementary file 1 [file Data_Sheet_1.pdf]

Individual Differences in Frequency and Topography of Slow and Fast Sleep Spindles

Roy Cox, Anna C Schapiro, Dara S Manoach, Robert Stickgold

Supplementary Results

Variability of channel-averaged N2 and N3 spectra

As reported qualitatively in the main paper (available at https://dx.doi.org/10.3389/fnhum.2017.00433), we observed large differences between N2 and N3 spectral profiles within individuals (Fig. 1A). To quantify this effect, we computed the degree of cross-stage similarity as the Pearson correlation between each individual's N2 and N3 channel-averaged spectral profiles. We did this separately for the two nights. Across subjects, this yielded moderate correlation values (night 1: 0.45 ± 0.27; one-sample t-test vs. zero: $t(27)=8.9$, $P<10^{-8}$; night 2: 0.50 ± 0.21; one-sample t-test: $t(27)=12.5$, $P<10^{-11}$). Additionally, we adjusted individual subjects' correlation P values for multiple comparisons using the False Discovery Rate (Benjamini and Hochberg, 1995), and found that 26/28 individuals had corrected P values <0.05 for night 1, and 24/28 for night 2.

However, these correlation values do not indicate whether spectra are more similar within than between individuals. To address this issue, we trained k-nearest neighbor classifiers on N2 power spectra and tested them on N3 spectra from the same night, and vice versa. Given chance level performance of 3.6% (1/28), resulting subject identification rates for classifiers trained on N2 and tested on N3 were modest at 18% (5/28, night 1) and 21% (6/28, night 2) (both $P<10^{-4}$). Performance for classifiers trained on N3 and tested on N2 was even lower: 7% (2/28, P=0.08, night 1) and 11% (3/28, P=0.017, night 2). This indicates that, with a few exceptions, spectral profile shapes during N2 and N3 were generally as different within as between individuals.

Stability of channel-averaged spectra across nights

We observed that spectral profiles are exceptionally similar across nights, including stable N2-N3 differences (Fig. 1C; Supplementary Fig. 1). To quantify this effect, we computed the cross-night similarity between night 1 and night 2 spectral profiles. We did this separately for N2 and N3. Across subjects, this yielded very high correlation coefficients of 0.97 ± 0.02 for N2 and 0.95 ± 0.05 for N3. These distributions were significantly different from zero (one-sample t-test: N2: $t(27)=215$, $P<10^{-44}$; 28/28 with $P_{corr}<0.05$; N3: $t(27)=215$, $P<10^{-35}$), and spectra were more similar across nights for N2 than N3 (paired t-test: $t(27)=2.8$, $P=0.009$; 28/28 with $P_{corr}<0.05$).

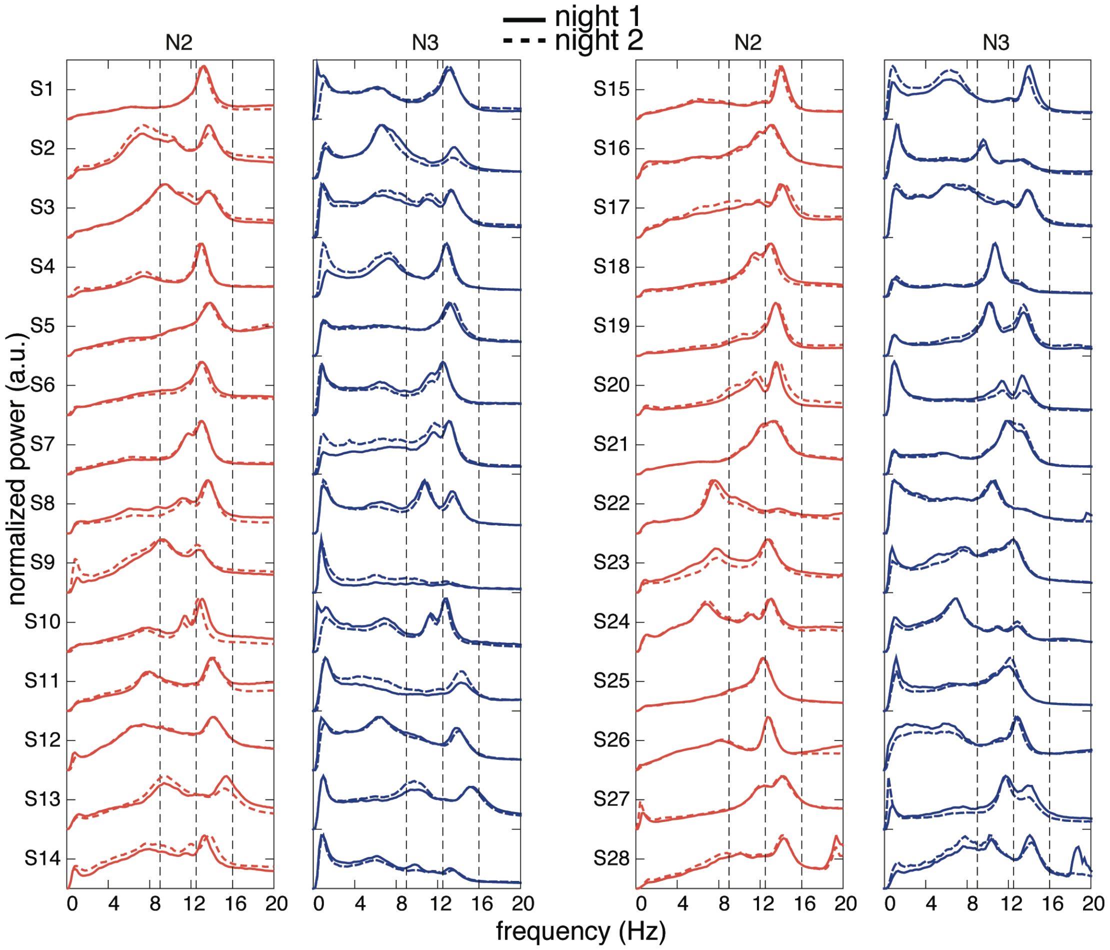

Supplementary Figure 1. Stability of channel-averaged N2 and N3 power spectra for all individuals across nights. All 28 subjects' (normalized and rescaled) N2 (orange) and N3 (blue) spectra are shown for night 1 (solid) and night 2 (dashed), demonstrating that individual differences in spectral shape are remarkably stable and constitute robust traits.

While these findings demonstrate high within-subject similarity of spectral profiles, we also observed quite high correlations between spectra from different individuals (N2: 0.71 ± 0.16; N3: 0.57 ± 0.21), indicating NREM spectra show lower but substantial baseline levels of similarity between subjects. To emphasize the fact that there is individual spectral stability beyond between-subject levels, we asked if we could identify individuals based on the similarity of their power spectra across nights. To this end, we trained classifiers on power spectra from one night and tested them on spectra from the other night, separately for N2 and N3. We obtained very high subject identification rates of 96% (27/28) for N2 (both directions: night 1->night 2 and night 2->night 1), and 82% (23/28) (night 1->night 2) and 89% (25/28) (night 2->night 1) for N3 (all $P<10^{-16}$, binomial test). This robust stability of NREM sleep spectra underscores the trait-like nature of individual differences in oscillatory expression.

Single-channel and component-based sigma frequencies

We directly compared sigma peak frequencies as determined from the single-channel and component approaches. Using frequency estimates averaged across the two nights, we obtained very high correlations with slopes close to 1 for fast sigma, during both N2 (R=0.93, $P<10^{-12}$, slope=0.92) and N3 (R=0.98, $P<10^{-17}$, slope=1.03). While we did see significantly higher peak frequencies for components relative to channels (paired t-tests, N2: t(27)=-2.2, P=0.03; N3: t(27)=-4,1, P=0.0003), the difference was only 0.1 Hz in both cases (component vs. channel, N2: 13.5 ± 0.6 vs. 13.4 ± 0.6 Hz; N3: 13.4 ± 0.6 vs. 13.3 ± 0.6 Hz). Thus, these findings indicate that the component analysis identified fast sigma peaks highly similar to those obtained from channels. As channel-based peaks could already be isolated from all 28 subjects, these findings also indicate that accurate fast sigma frequencies can be obtained without resorting to component decomposition. Nonetheless, these results offer an important degree of face validity to our approach.

In contrast, slow sigma showed much poorer correlations between channel- and component-defined spectral peaks during N2 (R=0.51, P=0.01) and N3 (R=0.57, P=0.002), with slopes substantially deviating from 1 (N2 slope: 0.42, N3 slope: 0.46). Peak frequencies of slow sigma activity did not differ significantly depending on detection method (paired t-tests, component vs. channel, N2: 10.9 ± 0.8 vs. 11.2 ± 1.0 Hz, t(22)=1.5, P=0.15; N3: 10.7 ± 0.6 vs. 10.7 ± 0.8, t(25)=-0.4, P=0.70), indicating that the component approach does not result in an overall shift of peak location.

Supplementary Discussion

All our analyses were performed on data transformed with the Laplacian operator (Perrin et al., 1989), which renders data reference-free and minimizes the effects of volume conduction, thereby making topographies more focal. Empirical and simulation studies suggest the Laplacian approach to result in enhanced sensitivity to subtle neurophysiological signals and to more accurately recover true underlying neural interactions (Cohen, 2014; Tenke and Kayser, 2015). This approach likely enhanced topographical variability in our data (e.g., between subjects, spindle classes, sleep stages), thereby achieving a major objective of this study (namely, to highlight such differences). Still, our group-level topographical analyses were highly consistent with conventional studies using spatially unfiltered data. We also assessed whether identified component peak frequencies depend on the Laplacian transformation. In several subjects, we performed the entire GED and peak detection procedure on data that was not Laplacian-filtered. However, detected peak frequencies were highly similar: slow and fast peaks detected in three subjects for both N2 and N3 showed identical frequencies for Laplacian and raw data for 11/12 comparisons, and the last one showed a 0.1 Hz difference. Altogether, these findings suggest the Laplacian approach offers an increase in effective spatial resolution without apparent drawbacks.

Of note, our GED implementation operates on covariance matrices obtained from band-pass filtered data in two pre-specified spectral ranges (9–12 Hz and 12–16 Hz).

We selected these frequency ranges based on visual inspection of channel-based spectra (Fig. 1 in main manuscript). However, we determined in several subjects that small shifts of 0.5 Hz of this initial slow/fast demarcation frequency did not affect the frequency of subsequently identified component peaks by more than 0.2 Hz, provided the subject's peak sigma frequencies fell in the selected ranges. Although the 9–12 Hz and 12–16 Hz spectral bands worked well in our sample of young healthy subjects, slow and fast spectral peak distributions in other samples might overlap more (Ujma et al., 2015), or might be clustered in different spectral bands in different age ranges (Shinomiya et al., 1999). In case no adequate group-wide division into spectral ranges can be found, we suggest a subject-specific approach may be used for identifying initial band-pass filtering ranges. Alternatively, an iterative approach could be employed where data is filtered repeatedly with slightly different slow/fast demarcation frequencies, which may then be used for subsequent GED and peak detection.

How many channels are needed to isolate slow and fast sigma components? Although high-density sleep recordings are becoming more common, smaller montages are still the norm. While a minimum of two channels is required numerically for GED computations, in practice one needs sufficient channels to capture the differential spatial expression of slow and fast spindles. Although we did not systematically investigate the effect of montage size, preliminary analyses in several subjects suggest that a montage of 19 channels may be sufficient, and successful component selection may even be possible with as few as five channels in some cases (Supplementary code available at https://doi.org/10.6084/m9.figshare.4905677). However, components based on fewer channels are more likely to reflect a mixture of slow and fast spindle activity, yielding less reliable estimates of slow and fast spindle frequencies. Indeed, whereas high-density recordings typically resulted in multiple components peaking in a very narrow (± 0.2 Hz) frequency range, components derived from smaller montages had more variable peak frequencies, making it more difficult to select the most appropriate component. Thus, high-density montages are recommended whenever possible.

Supplementary References

All cited works in this supplementary document are listed in the reference section of the main paper.
